# *De Novo* Assembly and Annotation of the Transcriptome of the Agricultural Weed *Ipomoea purpurea* Uncovers Gene Expression Changes Associated with Herbicide Resistance

**DOI:** 10.1534/g3.114.013508

**Published:** 2014-08-25

**Authors:** Trent Leslie, Regina S. Baucom

**Affiliations:** * Department of Biological Sciences. University of Cincinnati, Cincinnati, Ohio 45221; † Department of Ecology and Evolutionary Biology, University of Michigan, Ann Arbor, Michigan 48103

**Keywords:** resistance, transcriptome, RNA-seq, weed

## Abstract

Human-mediated selection can lead to rapid evolution in very short time scales, and the evolution of herbicide resistance in agricultural weeds is an excellent example of this phenomenon. The common morning glory, *Ipomoea purpurea*, is resistant to the herbicide glyphosate, but genetic investigations of this trait have been hampered by the lack of genomic resources for this species. Here, we present the annotated transcriptome of the common morning glory, *Ipomoea purpurea*, along with an examination of whole genome expression profiling to assess potential gene expression differences between three artificially selected herbicide resistant lines and three susceptible lines. The assembled *Ipomoea* transcriptome reported in this work contains 65,459 assembled transcripts, ~28,000 of which were functionally annotated by assignment to Gene Ontology categories. Our RNA-seq survey using this reference transcriptome identified 19 differentially expressed genes associated with resistance—one of which, a cytochrome P450, belongs to a large plant family of genes involved in xenobiotic detoxification. The differentially expressed genes also broadly implicated receptor-like kinases, which were down-regulated in the resistant lines, and other growth and defense genes, which were up-regulated in resistant lines. Interestingly, the target of glyphosate—EPSP synthase—was not overexpressed in the resistant *Ipomoea* lines as in other glyphosate resistant weeds. Overall, this work identifies potential candidate resistance loci for future investigations and dramatically increases genomic resources for this species. The assembled transcriptome presented herein will also provide a valuable resource to the *Ipomoea* community, as well as to those interested in utilizing the close relationship between the Convolvulaceae and the Solanaceae for phylogenetic and comparative genomics examinations.

The evolution of herbicide resistance in weedy plants is one of the most well-known examples of rapid, human-induced evolution and is of great interest in both basic and applied science. Herbicide-resistant weeds provide unique study systems for evolutionary genetics—a single dominant or codominant gene often controls the genetic basis of resistance (Warwick 1991), the selective agent is known and can be easily manipulated (Baucom and Holt 2010), and there are often multiple populations of the same herbicide-resistant weed present across the landscape such that the repeatability of any particular resistance mutation can be assessed (Delye *et al.* 2004). There is also a practical need for understanding the evolution of herbicide resistance. Present-day agriculture relies heavily on crops that are genetically engineered to be herbicide tolerant (Duke and Powles 2008a; Powles 2008), and, as a result, the use of one chemical on vast quantities of land has become commonplace (Gressel and Rotteveel 2000). Glyphosate, which is the active ingredient in the herbicide RoundUp (Monsanto, St. Louis, MO), is the prime example of this technology, and the use of this chemical for weed control has increased dramatically in agriculture in the last 20+ years, concomitant with the widespread adoption of transgenic, glyphosate-tolerant crops in 1996 (Baucom and Mauricio 2004). As a result of the increased reliance on this single herbicide in modern-day agriculture (Powles 2008), 24 weed species have developed glyphosate resistance (Heap 2011), and the inability to control glyphosate-resistant weed infestations is currently causing a significant negative economic impact on the agricultural industry (Duke and Powles 2008b).

Glyphosate resistance, in the handful of species that have been characterized, is controlled by target-site mutations, target-site expression alterations, and/or nontarget-site alterations (Shaner *et al.* 2012). Weed species such as Italian ryegrass, rigid ryegrass, and goosegrass exhibit a mutation in the herbicide’s target enzyme, EPSP synthase (Preston *et al.* 2009; Wakelin and Preston 2006). This mutation subsequently reduces the affinity of glyphosate for EPSP synthase, leading to less-competitive exclusion of EPSP synthase’s substrate (Duke and Powles 2008a). Palmer amaranth and common waterhemp both exhibit an increase in EPSP synthase expression, which compensates for the effects of glyphosate by producing more of the inhibited enzyme (Chandi *et al.* 2012; Gaines *et al.* 2010). The third and final type of genetic mechanism underlying glyphosate resistance involves nontarget-site alterations, that is, genetic changes involving a gene other than EPSP synthase. Nontarget site resistance has been hypothesized to occur in glyphosate-resistant horseweed and johnsongrass (Peng *et al.* 2010) and is thought to represent a putatively complex genetic mechanism(s) that may include genes involved in herbicide sequestration, oxidation, and other detoxification processes (Yuan *et al.* 2007). Interestingly, these genetic characterizations all use species that exhibit “evolved resistance,” which, in weed science, refers to a population of a once-susceptible species that evolved resistance after selection via the herbicide, generally in nature (Heap 2011). Because, by definition, this entails the use of species that have been exposed to potentially many generations of selection via the herbicide, the species in question may represent an upper evolutionary limit, *i.e.*, these studies assess the resistance phenotype after the fact of evolution rather than a controlled study of the process.

Some weed species, in contrast, exhibit low-level resistance to herbicide application (often called “tolerance” in weed science; *e.g.*, field bind-weed, velvetleaf); however, the genetic basis of this trait and its ability to respond to selection via the herbicide in such species virtually remain unexamined. *Ipomoea purpurea*, the common morning glory, is one such weed that exhibits low-level resistance to the field-rate application of RoundUp (Baucom and Mauricio 2008), and, morning glories in general have been implicated in a glyphosate-induced weed shift—the process whereby weed communities become dominated by a different group of weed species in response to the application of an herbicide (Culpepper 2006). We have previously investigated this low-level glyphosate resistance in *I. purpurea* using a quantitative genetics framework and uncovered genetic variation in resistance and positive selection on this defense trait in field conditions (Baucom and Mauricio 2008). We also have shown that variation in resistance will respond to artificial selection for increased and decreased resistance, and that it incurs a trade-off with plant growth and size (R. S. Baucom, unpublished data). However, we do not have an understanding of the genetic basis of resistance in this species, nor do we do know whether EPSP synthase is overexpressed in glyphosate-resistant lines as it is in palmer amaranth and common waterhemp.

The genetic characterization of glyphosate resistance in *I. purpurea* has been hindered by the fact that relatively few genomics resources exist for this species. The chloroplast genome of one *I. purpurea* accession has been fully sequenced (Mcneal *et al.* 2007), but in general the publicly available resources for this species are restricted to genes found in the flower color pathway (*e.g.*, Rausher *et al.* 1999; Zufall and Rausher 2003) and genes commonly used in phylogenetic examination (Miller *et al.* 1999). The lack of genomic tools for this species is surprising, because it has been used as a model since the late 1980s in ecological genetic studies that investigate the evolution of flower color polymorphisms, the evolution of the mating system, and the evolution of defense to herbivory (Baucom *et al.* 2011). In addition, the agriculturally prominent species sweet potato is in the morning glory genus (*Ipomoea batatas*)—and although a published transcriptome is available for sweet potato (Tao *et al.* 2012), its status as a hexaploid makes the use of this resource in the diploid, wild morning glories questionable. Increased publicly available genomic resources are thus needed in *I. purpurea* to expand and deepen ecological genetics investigations.

Here, and as part of our long-term goals to both expand genomic resources for *Ipomoea purpurea* and uncover the genetic basis of glyphosate resistance in this species, we present a fully annotated transcriptome and use this reference to examine the early gene expression differences of herbicide resistant selection lines after herbicide application. Whole-genome expression profiling has been used to study differential expression in *Drosophila* lines artificially selected for resistance to parasitoids, divergent behavior, and changes in locomotion (Edwards *et al.* 2006; Jordan *et al.* 2007), with the logic that transcripts that show consistent gene expression changes after artificial selection are candidate genes that affect the selected trait (Jordan *et al.* 2007). Although the use of whole-genome expression profiling can provide an unbiased estimate of which biological processes are differentially influenced by herbicide between selection lines, it represents, at best, an exploratory assessment that may or may not identify candidate loci involved in adaptation. Hence we use this technology to deepen our understanding of the gene expression changes that accompany direct and correlated changes after artificial selection for resistance to herbicide. The specific objectives of the work presented here are to (1) provide a thorough functional annotation to the proteins expressed in the *I. purpurea* reference transcriptome and assess the quality of the sequencing and assembly effort; (2) examine the prevalence and diversity of cytochrome P450 genes—known to provide a role in xenobiotic metabolism in plant genomes—within the *Ipomoea* transcriptome; (3) examine gene expression changes of artificially diverged herbicide resistant selection lines of *I. purpurea* using this reference transcriptome; and (4) validate gene expression differences using quantitative polymerase chain reaction (qPCR). This effort has resulted in assembled transcripts that will aide researchers in their goals to integrate knowledge of the phenotypic variation within this species with its underlying, causative genetic mechanism—and further, has provided candidate loci related to glyphosate resistance in the common morning glory for future investigation.

## Materials and Methods

### Experimental system

*Ipomoea purpurea* (L.) Roth (Convolvulaceae), the common or tall morning glory, often is found in corn, cotton, and soybean fields in the Southeast and Midwestern United States. Seeds of this species germinate mid-May through late August; flowering occurs 4-6 weeks post-germination, and individual plants are capable of producing as many as 8000 seeds per season. *I. purpurea* is an agricultural pest that is consistently listed by farmers as one of the “Worst Weeds in Agriculture” (Webster 2004; Webster and Coble 1997; Webster and MacDonald 2001).

Glyphosate [*N*-(phosphonomethyl)glycine] is the active ingredient in the nonspecific postemergence herbicide RoundUp. Glyphosate enters the plant by diffusion and moves into the plant phloem through either active or passive mechanisms (Shaner 2009). The herbicide translocates to the apical and root meristems, where it functions by competitively inhibiting EPSP synthase, a key enzyme in the shikimate pathway (Franz *et al.* 1997). This pathway is responsible for aromatic amino acids and secondary metabolites vital to plant growth and development (Tzin and Galili 2010).

### Sequencing of the reference *I. purpurea* transcriptome

To generate the *Ipomoea* reference transcriptome, a cDNA library was constructed using RNA extracted from stem, leaf, and bud tissue of a single *I. purpurea* individual grown from seed in ambient greenhouse conditions at the UGA Plant Biology Greenhouses in Athens, GA. This individual was generated by selfing a maternal line originally collected in 2002 from an agricultural field located in Watkinsville, GA. Tissues were collected, pooled, frozen with liquid nitrogen, and homogenized before shipment to BGI-Shenzhen (BGI), China, for RNA isolation. The specifics of RNA isolation, cDNA construction, and sequencing are presented in Johnson *et al.* (2012), and all steps were performed at BGI as part of a much-larger study that evaluates RNA extraction methods on transcriptome sequencing quality. To summarize, total RNA was extracted from pooled tissues using the Rneasy Plant Mini Kit (QIAGEN, Valencia, CA) and RNA purity, quality, and concentration were assessed using a NanoDrop8000 spectrophotometer (Thermo Scientific, Wilmington, DE) and the Agilent 2100 Bioanalyzer (Agilent Technologies, Santa Clara, CA). PolyA RNA was isolated from total RNA using the Dynabeads mRNA purification kit (Life Technologies, Grand Island, NY) and fragmented in a fragmentation buffer (Life Technologies) at 70° for 90 sec to 200−300 nt fragment sizes. Reverse transcription was completed using the SuperScript II reverse transcription kit (Life Technologies) and incubated with RNase H (Life Technologies) and DNA polymerase (Enzymatics, Beverly, MA). Short double-stranded cDNA was purified using the QIAquick PCR purification kit (QIAGEN), followed by end-repair using T4 DNA polymerase, Klenow polymerase, and T4 polynucleotide kinase (Enzymatics). Fragments of insert size 200 bp (±10% deviation) were gel purified and amplified by PCR using Phusion DNA polymerase (NEB, Ipswich, MA) and paired-end PCR primers resulting in a library of 322 bp long. Sequencing occurred in a lane of an Illumina GA IIx platform. The resulting sequence reads were filtered prior to scaffold assembly to ensure that paired reads did not have >5% uncalled bases (Ns), 20 bp of low-quality bases (Phred-type Q-score <10), reads with >15 bp of continuous adapter sequence, and more than 0- or 1-bp mismatch in a 6-bp index sequence (used during library creation to differentiate samples). These four criteria were implemented to eliminate contaminant sequences and to ensure that only high quality reads were used in subsequent assemblies. The *Ipomoea* transcriptome is available through the 1kp plant transcriptome project and can be accessed through http://www.onekp.com/. Annotation of the *I. purpurea* reference transcriptome is made available as Supporting Information and can be found in Table S1.

### *De novo* assembly and assessment of transcriptome completeness

The transcriptome was assembled using SOAPdenovo-Trans (http://soap.genomics.org.cn/SOAPdenovo-Trans.html), which is designed to assemble large transcriptomes using sequences from short-read sequencing platforms. The parameters used to produce the assembly were as follows: –K 25 (25-mer size), –L 100 (minimum contig length used for scaffolding), –e 2 (delete contigs with coverage less than or equal to 2), and –t 5 (output no more than five transcripts from each locus). We assessed the completeness of the transcriptome in two ways. First, we determined how many of our assembled transcripts were represented by full-length proteins by predicting open reading frames using the program OrfPredictor (Min *et al.* 2005) followed by a blastp (e^−20^) of the predicted peptide sequences to a database of plant proteins (tomato, Arabidopsis, maize, rice) downloaded from National Center for Biotechnology Information (NCBI). Second, we used the program Core Eukaryotic Genes Mapping Approach (Parra *et al.* 2007) to determine how many of 248 highly conserved eukaryotic genes were present in the *I. purpurea* transcriptome. Finally, we estimated the abundance of each transcript using the Trinity program align_and_estimate_abundance.pl, which uses Bowtie v1.0.1 to align reads (Grabherr *et al.* 2011). RSEM (Li and Dewey 2011) was then used to generate transcript percentages and fragments per kilo bases of transcript per million mapped reads (FPKM) values for relative expression of each transcript.

### Functional annotation of the reference transcriptome

We annotated assembled sequences for function using the NCBI blastx program (v2.2.17; Altschul *et al.* 1997) in separate blasts using the nr database from NCBI (e^−3^). The XML formatted output from the blast to NCBI’s nr database was loaded into Blast2GO (v2.6.6; Conesa *et al.* 2005), and scaffolds with a significant blast hit were annotated for molecular function, biological process and cellular component using both Gene Ontology (GO) and GO Slim annotations. We examined the potential that transcripts may include transposable elements by performing a blastn (e^−10^) analysis using a database of transposable elements developed from randomly sampled *Ipomoea purpurea* genome sequence and the program Assisted Automated Assembler of Repeat Families (*i.e.*, AAARF; Debarry *et al.* 2008; R. S. Baucom and J. Bennetzen, unpublished data). The original reads used for assembly of this transcriptome and the assembled scaffolds are publicly available on the European nucleotide archive (1000 Plants transcriptomes; Project ID PRJEB4922, Sample accession SAMEA2242471) and the 1000 Plants (1kp) pilot website, respectively (http://mirrors.iplantcollaborative.org/browse/iplant/home/shared/onekp_pilot). Annotations of this transcriptome using blast and Blast2GO are made available as supplementary information to this manuscript (Supporting Information, Table S1).

### Analysis of transcripts encoding cytochrome P450 genes

Transcripts that were annotated as cytochrome P450 genes were examined further to determine how many members of this large gene family were successfully sequenced in the *I. purpurea* transcriptome. We extracted the transcripts identified from the blastx to the NCBI nr database as cytochrome P450 and performed a blastx (e^−10^) with a database of curated tomato cytochrome P450 proteins downloaded from http://drnelson.uthsc.edu/tomato.html, which included both genes and pseudogenes. For visualization of blastx results, we imported the top tomato P450 hit of each of our transcripts, along with the bit score of the blast alignment to the program Cytoscape (P. Shannon 2003). Additionally, we assessed the quality of these transcripts by determining how many cytochrome P450s were represented by full-length proteins after predicting open reading frames (ORFs) and performing alignments with plant proteins (as presented previously).

### Germplasm collection, generation of selection lines, and preparation for RNA-seq

**Figure 1:**
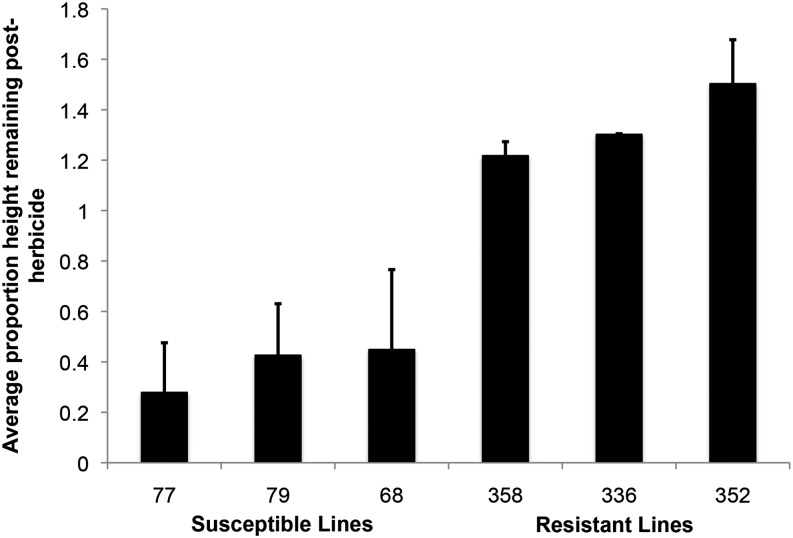
The average proportion of the height remaining of the main stem after an application of glyphosate (0.8 kg a.i./ha) of the least- (Susceptible) and most-resistant (Resistant) *I. purpurea* families. Bar height represents family average, and SEs were calculated using three treated individuals per family.

We performed two generations of artificial selection using parents sampled from the same base population, initially collected in 2000 in Watkinsville, GA, to both increase and decrease glyphosate resistance in this species (detailed in R. S. Baucom, unpublished data). A progeny resistance assay after the second generation of artificial selection identified the three most/least resistant families from the R and S selection lines as the families that, on average, exhibited the greatest and least proportion height remaining following herbicide application (Figure 1). Replicates of the three R lines used in the RNA-seq survey exhibited 1.34 ± 0.084 (average ± SE) proportion height remaining of the main stem 3 wk after the herbicide application, whereas replicates of the S lines exhibited 0.38 ± 0.05 proportion height remaining (Figure 1). Thus, the R lines used in the transcriptomic survey of gene expression were capable of continued growth after herbicide application, whereas the S lines significantly died back in response to the herbicide.

To prepare RNA for transcriptome sequencing, full-sibling replicate seeds from six families —three families each of those identified as the most/least resistant from the R/S selection lines were planted in 4-inch pots with Fafard 3B soil (Agawam, MA) and randomized in the greenhouse on June 3, 2012. Plants were sprayed with 0.8 kg ai/ha of RoundUp on June 20, 2012, with a pressurized CO_2_ backpack plot sprayer (R & D Sprayers, Opelousas, LA) at the 4−5 leaf stage. Leaf material from each plant was collected 8 hr after herbicide application, snap frozen in liquid nitrogen, and stored at −80°. In this study we chose to focus on potential differences in early response to glyphosate application since preliminary data (*not shown*) indicated that susceptible lines began shutting down gene expression rapidly following herbicide application. RNA-seq data are available in the SRA database of NCBI and can be accessed via this link: http://www.ncbi.nlm.nih.gov/bioproject/?term=PRJNA216984.

### Preparation of cDNA for RNA-seq of experimental individuals

Total RNA was extracted from experimental individuals using the QIAGEN Rneasy Plant Mini Kit (Hilden, Germany). Each RNA extraction contained 100 mg of leaf tissue ground up in liquid nitrogen using mortar and pestle. Duplicate RNA extractions from R and S individuals were prepared and treated with DNAse I to remove genomic DNA contamination. Duplicates were then pooled according to individual and the RNA from three herbicide treated R and three S families was sent to Purdue Genomics for library preparation and sequencing on the Illumina HiSeq2000 System. RNA quality and concentration was measured on a Nanodrop UV spectrophotometer and an RNA Nano chip on an Agilent Bioanalyzer 2100. Six separate libraries were prepared from total RNA using the Illumina TruSeq RNA kit. Library preparation followed the manufacturer’s instructions, and the libraries were pooled using adapter indices supplied by Illumina (barcodes AD012, AD013, AD014, AD015, AD016, and AD018). Sequencing was performed in a single lane on an Illumina HiSeq2000. The samples were sequenced as 101-bp, paired-end reads.

### Quality filtering, mapping of sequence reads, and analysis of differentially expressed genes

Adapters were trimmed from sequence reads using trimmomatic (v. 0.22). Reads that did not pass a quality threshold score of 20—those with average nucleotide misread probabilities below 1%—were removed using the default Bioscope (v1.3) parameters. Paired-end filtered reads were then mapped to the reference *I. purpurea* transcriptome (described below) using Bowtie2 (v2.1.0). Minimum and maximum insert sizes were set at 150 bp and 350 bp, respectively, to account for variation in insert size within and across samples. Alignments that did not have a successfully aligned mate within the acceptable insert size range were discarded as were reads that did not uniquely align to a single scaffold. Detailed mapping parameters and mapping efficiencies are described further in Table S2 and Table S3. Raw gene counts were determined using htseq-count, a function from the Python module HTSeq (v. 0.5.4p1), with the uniquely aligned paired-end reads. Scaffolds with a minimum of 10 raw counts in at least two libraries were considered high-confidence transcripts for analysis. Differential expression was investigated using the edgeR (Robinson *et al.* 2010) package in R (R Core Team 2013), which implements a negative binomial distribution for the modeling of gene expression and uses the trimmed mean of M-values approach (Robinson and Oshlack 2010) for calculating a mean for normalization. *P*-values were adjusted using edgeR’s default Benjamini-Hochberg procedure. We examined both the position on the scaffold to which the reads mapped and the scaffold length to determine whether they might influence our ability to assess differences in gene expression between resistant and susceptible lines. We uncovered weak evidence that mapping position and transcript length influence expression across transcripts (Figure S1), and present an analysis that shows these expression differences do not impact our ability to identify differentially expressed genes (DEGs) between our R and S families in Figure S2 and Table S4.

### Validation of expression using qPCR

We used qPCR to validate the gene expression profiles from the RNA-seq experiment. RNA extracted from herbicide treated R and S replicates was reverse transcribed using the Roche Reverse Transcription High-fidelity cDNA Synthesis Kit (Penzberg, Germany). The same six individuals that were submitted to Purdue Genomics for Illumina sequencing were used in the qPCR confirmation. All qPCR sample reactions were performed in duplicate, and all six RNA-Seq samples were run on the same plate for each gene. The control gene, glyceraldehyde 3-phosphate dehydrogenase (GAPDH; F: 5′ ACACTTTGGAGGAAGAAGGA 3′; R: 5′ GTTATTGCCTGGTACGACAA 3′, amplicon length, 188 bp, melting point, (Tm) ~53°) was designed using Primer3 on annotated scaffolds from the reference *I. purpurea* transcriptome and selected as our control based on primer efficiencies and consistent Cq values obtained across samples. Furthermore, the cDNA was kept constant across all qPCR samples (100 ng), allowing for comparison of mean GAPDH Cq values between resistant and susceptible individuals.

Fourteen genes were selected for validation by qPCR based on the specificity of their top blast description and previous association with glyphosate resistance in other weed species. Scaffolds that had a top blast describing a cytochrome P450, glutathione S-transferase, or ABC transporter gene were included in the qPCR confirmation if they had a significant *P*-value before correction in the RNA-seq gene expression analysis (only one transcript selected with this method survived p-value correction). All primer sets were designed with Primer3 on the annotated reference transcriptome scaffolds and tested for amplification by standard PCR. Primer sequences and characteristics of these loci are available in Table S5. Primer efficiencies were calculated directly from qPCR fluorescence curves by the software LinRegPCR (Ramakers *et al.* 2003; Ruijter *et al.* 2009). To normalize relative mRNA abundance among samples, qPCR results from DEGs of interest were compared to the control gene GAPDH. This comparison was accomplished by finding the difference between the mean Cq value of the DEG and the mean Cq value of GAPDH. The respective Cq values were determined by the PCR cycle at which the sample product reached the fluorescence value of 0.2.

## Results

### Reference transcriptome quality and completeness

**Figure 2:**
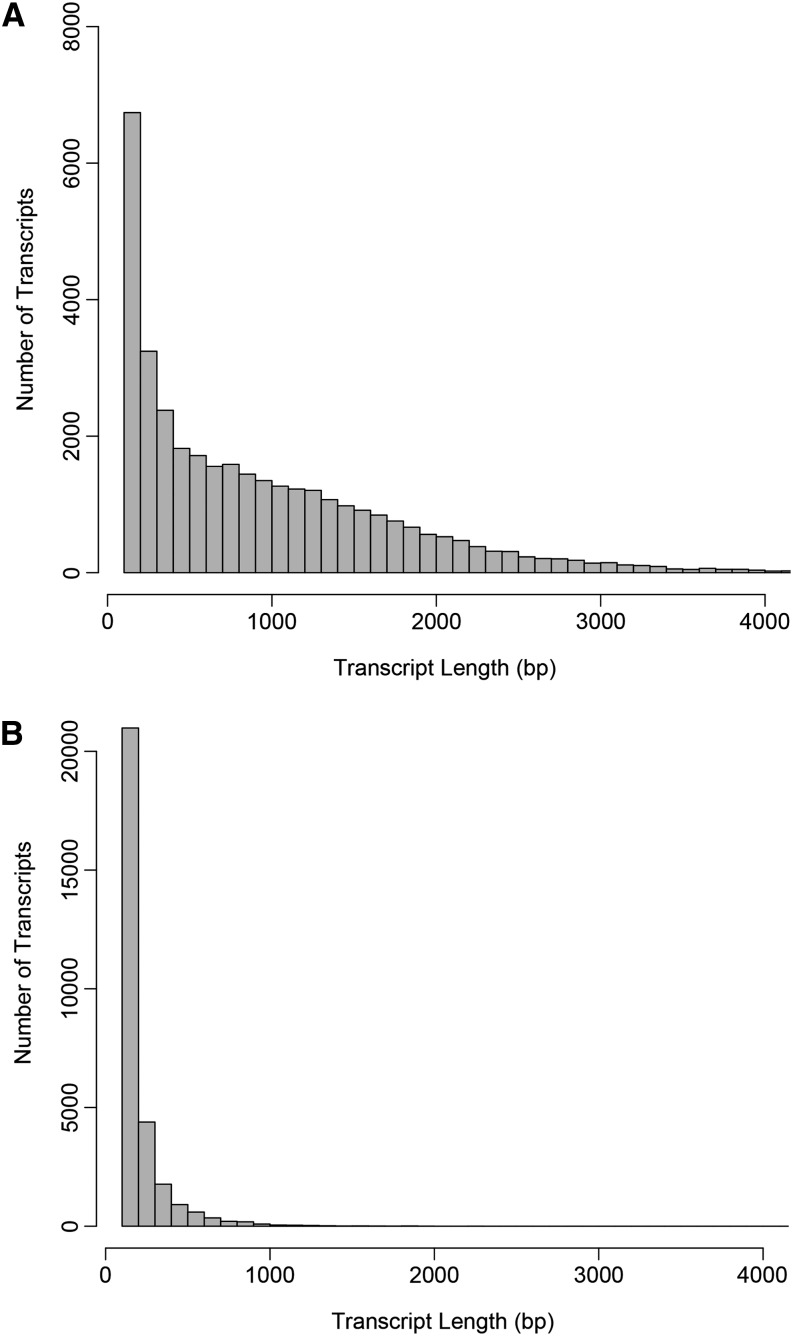
Transcript size distribution shown by (A) transcripts that were annotated by blastx to the National Center for Biotechnology Information nr database, and (B) transcripts that could not be annotated by blast.

Sequencing on the Illumina GA IIx platform generated 13.6 M 75 bp paired-end sequence reads for a total of 1.85 Gb of high-quality bases. Assembly of these reads using SOAPdenovo-Trans returned 64,956 scaffolds. Approximately 46% of the assembled transcripts were <300 bp; 54%, or 35,297 were ≥300 bp (Table 1 and Figure 2). The average length of transcripts greater than or equal to 300 bp was 1142.4 bp whereas the maximum length of transcripts was 9192 bp (Table 1). The N50 value of all transcripts was 1339 bp. Of the 13.6 M total reads sequenced, we were able to map the majority of reads, 85.6%, back to the transcripts using bowtie.

**Table 1:** Assembly statistics

	*I. purpurea* transcriptome
Raw sequencing reads	13,087,472
Number of quality Q20 bases, Gb	1.86
Number of transcripts, total	64,957
N50, bp	1339
Number of transcripts, <300 bp	29,660
Number of transcripts, ≥300 bp	35,297
Average length, bp, ≥300 bp	1142.4
Median length, bp, ≥300 bp	918
Maximum length, bp, ≥300 bp	9192

**Figure 3:**
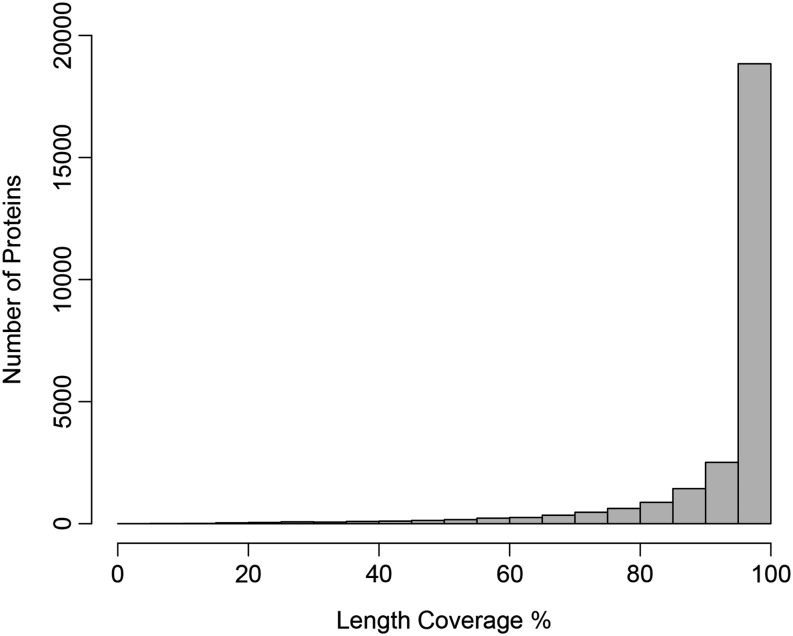
Protein length coverage of translated sequences in the *I. purpurea* transcriptome compared with a database of maize, rice, tomato, and Arabidopsis proteins.

We evaluated the quality and completeness of the *I. purpurea* transcriptome by assessing the number of core eukaryotic genes that were present among transcripts and by determining the percentage of full-length proteins that were sequenced. The Core Eukaryotic Genes Mapping Approach program identified a total of 240 of the 248 core eukaryotic genes, and 220 of these were considered complete (>70% of the protein identified). We next predicted open reading frames with the program OrfPredictor and performed a blastp (e^−20^) with a database of plant proteins (tomato, Arabidopsis, maize, rice) using the predicted peptide sequences. OrfPredictor predicted 63,857 open reading frames, leaving 1099 transcripts that did not return a potential ORF. Of these predicted ORFs, 29,615 returned a significant blastp hit to our database of plant proteins, and 21,371 of these sequences exhibited >90% alignment coverage (Figure 3). Thus, ~46% of the predicted peptide sequences in the *I. purpurea* transcriptome were nearly full-length.

### Functional annotation of the reference transcriptome

Of the 64,956 assembled transcripts, 35,282 (54%) exhibited sequence homology to a sequence within NCBI’s nr database (Table 2), leaving 29,667 transcripts that could not be annotated. The majority of the transcripts without a blast hit were short (<300 bp in length), with >20,000 of these transcripts being very short, *i.e.*, between 100 and 200 bp (Figure 2B). Approximately 6000 short transcripts—those between 100 and 200 bp in length—were identified through blastx (Figure 2A). We further investigated expression of annotated and nonannotated transcripts according to sequence length. Transcripts that exhibit homology to a sequence within the nr database show greater average expression compared to transcripts that could not be annotated [Figure S3A, Average (± SD) FPKM of annotated transcripts: 27.7 (±10.4); average (± SD) FPKM of nonannotated transcripts, with three outliers removed: 18.3 (±21.9), Wilcoxon two sample test W= 375, *P* = 0.002]. The nonannotated transcripts exhibit a wide range of expression values according to transcript length (Figure S3B) whereas, in general, the annotated transcripts exhibit some, but markedly less variation in expression according to sequence length (Figure S3A). Such a result is a consequence of slightly fewer nonannotated transcripts compared with annotated transcripts, and effectively 0 expression for the 100- to 200-bp nonannotated transcripts as well as more sequence length bins that exhibit, on average, very low or close to zero expression in the nonannotated set. The wide variation in expression values for the nonannotated set of transcripts likely reflects that many of these transcripts are assembly errors rather than functionally transcribed genes. It is also possible that the nonannotated transcripts are sequenced introns stemming from DNA contamination in the sample. Regardless, we elected to retain all transcripts in our downstream analyses given that some of these transcripts *could* be biologically functional.

**Table 2:** Annotation statistics

	All Sequences
Total number of sequences	64,956
Sequences without BLAST hits	29,667
Sequences with BLAST results	2164
Sequences with mapping results	4962
Sequences with GO annotations	28,156

GO, gene ontology.

We further screened the transcripts for significant identity to transposable elements using a database of TE sequences mined from *I. purpurea* genomic DNA. To consider a transcript as an expressed, sequenced transposable element, we used a relatively strict e-value (e^−10^) in a blastn, and required that significant hits exhibit an alignment length of at least 100 bp. Using these parameters, we recovered 476 transcripts that exhibited homology to a TE sequence. The majority (60%) of these transcripts exhibited homology to repetitive elements within our database that remain nonannotated and thus novel—a result that is not surprising given the high variability in TE families in other plant genomes and the difficulty they pose for the annotation process (Wicker *et al.* 2007). Of the identified TE sequences, ~28% were Class II or DNA elements whereas ~10% were Class I retroelements. Of the identified DNA elements among our transcripts, we found representatives of the Ac/Ds, Mu and MuDR, CACTA, EnSpm, and Helitron super-families (Table 3). We also identified the superfamilies *Copia* and *Gypsy* among the transcripts that were identified as Class I transposable elements (Table 3).

**Table 3:** Summary of repetitive elements identified in the *I. purpurea* transcriptome

Element Type	Number of Transcripts
Class II elements	
Ac/Ds	17
MuDR	3
Mu	2
CACTA	34
EnSpm	19
Unknown repeat, likely DNA element	46
Helitron	14
Class I elements	
Copia	20
Gypsy	22
unknown retro	7
Unknown repetitive element	286
rRNA	6
Total	476

**Figure 4:**
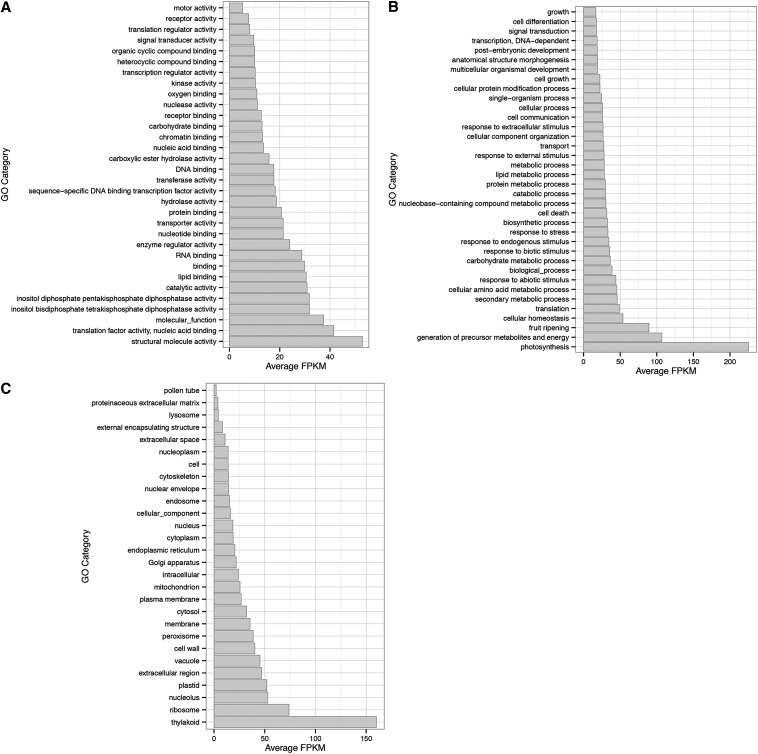
Average expression (fragments per kilo bases of transcripts per million mapped reads [FPKM]) values according to Gene Ontology (GO) Slim categories within the (A) Molecular Function, (B) Biological Process, and (C) Cellular Component GO categories.

Following assignment of the top blast hit via blastx and the screen for TE sequences, we loaded the xml formatted blast results into the Blast2GO program to assign GO terms and performed further annotation of the transcripts. Of the 33,118 transcripts with at least one GO-term assignment, 28,156 were annotated in the level 1 GO categories of biological process, molecular function, and cellular component. The GOSlim category assignments of transcripts are presented in Figure S4, and the overall assignments are available in the supplemental annotation file (Table S1). The GOSlim categories that exhibit highest expression, on average, in the *I. purpurea* transcriptome are associated with photosynthesis (Figure 4) (structural molecule activity (Figure 4A), photosynthesis and generation of precursor metabolites (Figure 4B) and thylakoid (Figure 4C)—this result is in line with our expectations since the majority of the plant tissue used for RNA extraction was leaf tissue. Flower bud and stem tissues were also included in this bulk sample and as a result we were able to recover expressed transcripts that belong to, for example, the ‘pollen tube’ GOSlim category (Figure 4C).

### Cytochrome P450 gene family

**Figure 5:**
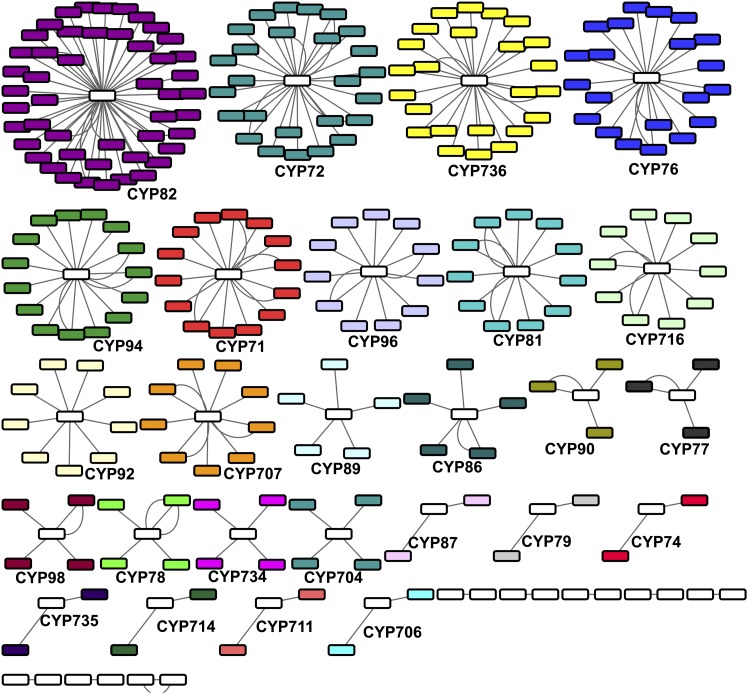
The cytochrome P450 genes identified in the *I. purpurea* transcriptome by a blast analysis using the cytochrome P450 family and subfamily sequences from tomato. Each circle, or node, represents a transcript from *I. purpurea*, with the tomato cytochrome P450 families indicated by separate colors.

The cytochrome P450gene family is one of the largest families in plant genomes with as many as 300 genes belonging to 50 gene families (Nelson *et al.* 2004; Hamberger and Bak 2013). Given the diversity of functions that cytochrome P450 genes perform in plant genomes, we elected to explore the number of cytochrome P450 genes, and their assembly quality in the *I. purpurea* transcriptome to provide further context for expression patterns detected in our RNA-seq screen (*presented below*). Of the ~35K transcripts in the *I. purpurea* transcriptome that exhibited a significant blast hit, 289 were identified as a cytochrome P450 gene, and a blastx to a curated database of tomato cytochrome P450 proteins further identified 34 families and 121 subfamilies of P450 gene within *I. purpurea*. Families homologous to the tomato cytochrome P450 gene families, and their relative sizes in the *I. purpurea* transcriptome, are shown in Figure 5. Of these 121 subfamilies, 7 exhibited homology to subfamilies that are pseudogenes in tomato (Table S6). Seven transcripts did not exhibit homology to a curated tomato P450 protein and could not be assigned to a family. The CYP82 superfamily is the most highly represented P450 gene family in the *I. purpurea* transcriptome, with 53 transcripts belonging to seven different subfamilies (Figure 5). The sequencing and assembly effort presented herein thus produced a large number of P450 genes for downstream analysis, and the majority (~250) of these transcripts were sequenced at almost the full length of other plant cytochrome P450 proteins (*i.e.*, >90% length; Figure S5).

### Illumina sequencing of glyphosate-resistant selection lines and mapping to the reference transcriptome

Nearly 96% of reads (442 of 462 million raw reads) from the Illumina HiSeq2000 Sequencing System passed Bioscope (v1.3) quality filters (details in Table S2 and Table S3), and on average, 73.7 million high-quality reads were processed per sample (range: 39.0−146.9 million). To identify the gene from which sequence reads originated, high-quality reads were mapped against the *I. purpurea* reference transcriptome presented herein. On average, 15.8 million paired-end reads per sample mapped uniquely to the reference transcriptome (range: 7.6−28.7 million). Thus, the Illumina sequencing yielded a gross mapping efficiency of 40.8% with the raw paired-end reads and a net mapping efficiency of 42.8% with the paired-end high-quality reads. Our overall mapping efficiency is similar to comparable studies, which report 23.7% mean gross mapping efficiency and 42.7% mean net mapping efficiency (Meyer *et al.* 2011).

The majority of the cDNA sequences in our reference transcriptome were detected in our RNA-seq dataset. For example, the Illumina sequence reads mapped to 20,622 (96.3%) of the 21,416 scaffolds from the reference transcriptome that matched known protein sequences. 20,001 (97.0%) of these reference cDNA sequences had a minimum of ten paired-end reads that aligned in at least two of our samples; we considered these scaffolds our high-confidence scaffolds and retained them for gene expression analysis. Of the 20,001 reference scaffolds, 19,921 (99.6%) matched known proteins, and 80 (0.4%) matched unknown or hypothetical proteins. Only 11 (0.05%) matched nuclear-encoded rRNA genes, 296 (1.5%) matched mitochondrial-encoded genes, and 499 (2.5%) matched chloroplast-encoded genes; this suggests negligible contribution from non-coding RNA and organelle-derived genes.

### Comparative gene expression between selection lines

**Figure 6:**
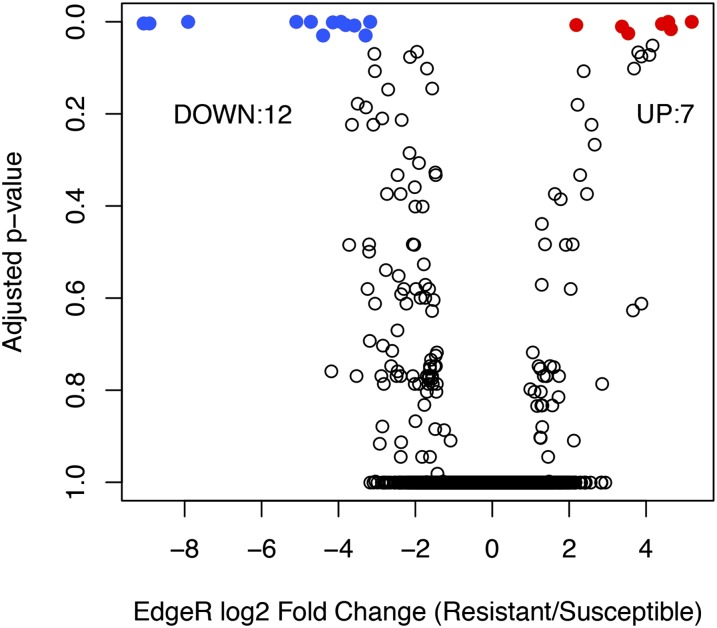
edgeR-adjusted *P* values plotted against edgeR log2 fold changes. Note the ratio is R to S (positive ratios indicate increased expression in R lines, and *vice versa*).

**Figure 7:**
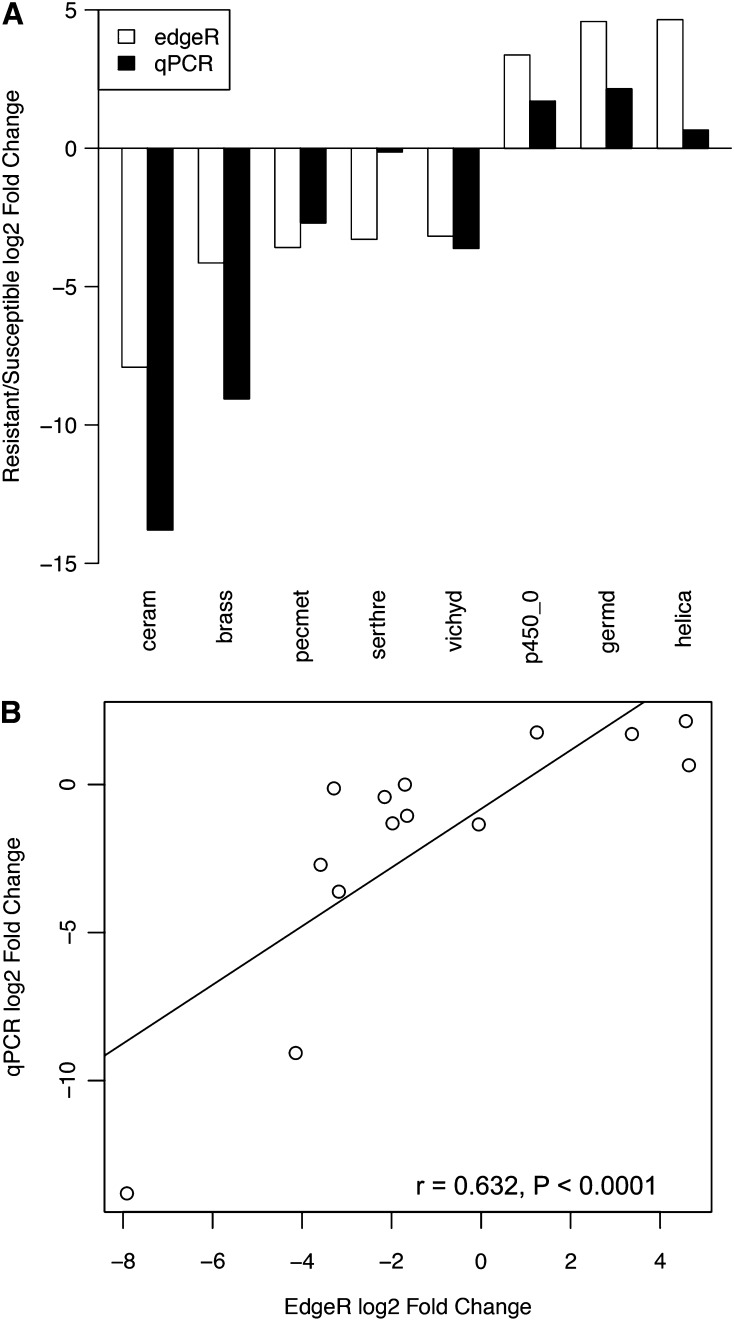
Confirmation of RNA-seq results. (A) Bar plot of edgeR and quantitative polymerase chain reaction (qPCR) log2 fold changes from resistant to susceptible lines (positive fold change indicates greater expression in resistant lines). Top blast descriptors are as follows: ceram is ceramidase family protein, brass is brassinosteroid insensitive 1−associated receptor kinase 1, pecmet is pectin methylesterase, serthre is receptor serine-threonine protein, vichyd is vicianin hydrolase-live, p450_0 is cytochrome p450 82a3-like, germd is (-)-germacrene d synthase, and helica is u5 small nuclear ribonucleoprotein helicase. B) Confirmation of 14 edgeR differentially expressed genes by qPCR (r = 0.632, *P* = 0.000675).

Analysis using edgeR detected 19 genes that were differentially expressed between resistant and susceptible individuals following *P*-value adjustments (Figure 6). Twelve transcripts were down-regulated, and seven transcripts were up-regulated in the resistant compared to susceptible families (Figure 6). Seven of these were annotated by Blast2GO as simply “protein” (Table 4). Five of the seven transcripts that were up-regulated in the resistant families could be annotated—one exhibited homology to a member of the cytochrome P450 family, two were germacrene D-synthases, one a protein kinase, and the final up-regulated transcript exhibited homology to a ribonucleoprotein helicase (Table 4). Seven of the 12 transcripts that were down-regulated in resistant compared to susceptible families were annotated as a viacianin hydrolase-like protein, a pectin methylesterase, a ceramidase family protein, an ATP-binding protein, and a brassinosteroid insensitive 1-associated receptor kinase (Table 4). There was no evidence that the scaffold identified as EPSP synthase exhibited differential expression between the R and S lines (edgeR Log Fold Change = 0.013, *P*-value = 0.74) indicating that resistance in this species is not conferred by overexpression of EPSP synthase as in other species. Finally, the qPCR analysis of the expression patterns of 14 genes support our RNA-seq results (Figure 7). The expression patterns of transcripts identified as differentially expressed exhibit the same patterns in the qPCR validation (Figure 7A); over all loci tested, the relationship between the two methods was positive and significant (r = 0.632, *P* < 0.001, Figure 7B).

**Table 4:** edgeR differentially expressed genes

Scaffold ID	Top Blast	logFC, Fold Change	logCPM, Counts per Million	*P* Value	Padj.
2055131	Protein (*XP_002316618*)	−9.06	−0.34	1.44E-06	3.19E-03
2011172	Cysteine-rich receptor-like protein kinase 10-like	−8.91	−0.47	1.64E-06	3.29E-03
2056577	Ceramidase family protein (*XP_002520446*)	−7.91	0.59	9.29E-10	6.19E-06
2010370	atp binding (*XP_002518555*)	−5.1	2.91	2.18E-08	7.28E-05
2009240	Protein (*ACM89479*)	−4.72	3.31	2.70E-14	5.41E-10
2004378	Protein(*CBI29180*)	−4.4	−0.33	2.79E-05	2.94E-02
2061274	Brassinosteroid insensitive 1-associated receptor kinase 1	−4.14	3.06	2.54E-07	6.36E-04
2009241	Protein (*XP_002304944*)	−3.93	2.47	4.60E-09	2.30E-05
2004377	Protein (*CBI29180*)	−3.81	2	4.67E-06	7.18E-03
2017152	Pectin methylesterase (*CAD29733*)	−3.58	3.01	5.63E-06	8.04E-03
2055046	Receptor serine-threonine protein	−3.29	1.91	2.75E-05	2.94E-02
2063945	Vicianin hydrolase-like (*XP_002330884*)	−3.18	4.32	8.64E-09	3.46E-05
2002437	Protein (*XP_002285664*)	2.18	4.04	3.80E-06	6.33E-03
2003581	Cytochrome p450 82a3-like (*XP_002282035*)	3.37	1.13	8.05E-06	1.07E-02
2054556	Protein (*XP_002312901*)	3.54	0.41	2.14E-05	2.51E-02
2059855	Protein kinase	4.41	2.48	2.30E-06	4.19E-03
2001731	(-)-germacrene d synthase (*AAR99061*)	4.58	2.56	3.10E-10	3.10E-06
2013762	u5 small nuclear ribonucleoprotein helicase (*XP_002266580*)	4.65	0.02	1.29E-05	1.62E-02
2005233	(-)-germacrene d synthase (*AAS66357*)	5.18	1.12	4.61E-08	1.32E-04

Note logFC is a resistant/susceptible ratio.

## Discussion

Here we present the first functionally annotated transcriptome of the common morning glory, *Ipomoea purpurea*, which is a model species in evolutionary ecology and ecological genetics. High-throughput sequencing of RNA extracted from stem, leaf, and flower bud tissue of this species followed by a *de novo* transcriptome assembly produced a dataset of ~65,000 transcripts. More than 33,000 sequences were annotated by blast and mapped with at least one GO category; GO categories that exhibited the greatest average expression in this transcriptome were those involved with the photosynthetic process. We examined the quality of the transcriptome assembly in terms of the length of the proteins assembled and their expression patterns. Many of the sequences were assembled near the full-length of plant proteins from other species, and further, we identified a large number of cytochrome P450 genes that were also assembled near full-length. We also present an RNA-seq survey designed to detect gene expression changes of resistant and susceptible *I. purpurea* lines upon early challenge with the herbicide glyphosate. We uncovered a handful of transcripts that were differentially regulated and verified the gene expression patterns by using qPCR. Herein, we expand upon the ecological study systems that will benefit from the annotated transcriptome and place the differences in gene expression between selection lines in the context of the hypothesized mechanism of resistance in *I. purpurea*.

### Functional annotation of the *de novo I. purpurea* transcriptome

The transcriptome assembled here will provide basic genomic tools that will enrich the study of herbicide resistance evolution as well as add to a variety of ecological genetics and evolutionary ecology investigations. For example, we annotated 124 ABC transporters, which are transmembrane proteins involved in transporting substances across extra- and intracellular membranes that have been indicated in the development of herbicide resistance in *Conyza* species (Peng *et al.* 2010). A search of the transcripts for the top blast hit containing the term ‘myb’ returned approximately 98 MYB transcription factors—these genes are part of a large gene family (~130 members) that influence floral pigment intensity and could thus be useful in the investigation of the evolution of the floral color pathway (Martin and PazAres 1997). We further sequenced and annotated ~10 transcripts that could be useful in mating system examinations—*e.g.*, transcripts identified as “pollen-specific proteins” as well as a ‘self-incompatibility locus.’

Because we used non-normalized RNA library for the reference transcriptome, we were able to assess the expression patterns of the transcripts according to both sequence length and annotation status. We were further able to assess the relative expression level according to GO assignment. In general, we found that numerous very short transcripts could not be annotated by blast, and these transcripts were also generally not expressed—thus, such transcripts are most likely assembly errors rather than transcribed genes. Further, we found that out of the ~65,000 total assembled transcripts, 54% exhibited homology to a protein within the nr database, and ~43% were annotated by GO categories—these findings is similar to many other plant transcriptome annotations, such as tobacco, sweet potato, and dodder (Liu *et al.* 2013; Nakasugi *et al.* 2013; Ranjan *et al.* 2014; Tao *et al.* 2012). However, if we were to have excluded the approximately 29,600 transcripts that were short, nonannotated, and exhibited low expression values, our annotation success would be closer to 78%. Regardless, the transcriptome presented here is of sufficiently high quality to provide significant advancement in the genomic resource for this species, since nearly half of the predicted ORFs present in this transcriptome were sequenced close to 100% the length of proteins from other plant species.

One of the aims of this work was to assess the number and quality of cytochrome P450 transcripts identified within the *I. purpurea* transcriptome. The cytochrome P450 gene family is one of the largest gene families within plant genomes, containing as many as 300 copies that belong to a diverse group of sub-families (Nelson and Werck-Reichhart 2011). The cytochrome P450s are broadly indicated in a variety of plant metabolism pathways—because they catalyze the biosynthesis of specialized plant compounds, the P450 genes play a pivotal role in the production of plant chemistry (Hamberger and Bak 2013). Plant terpenoids, phenylpropanoids, and nitrogen-containing compounds—which include alkaloids, cyanogenic glucosides and glucosinolates—are crucial compounds that provide chemical defense from herbivores, UV protection, pigmentation, and structural components of membranes, among other functions (Hamberger and Bak 2013). We chose to investigate their diversity and sequence quality in the *I. purpurea* transcriptome since we uncovered a P450 transcript that is overexpressed in the herbicide-resistant selection lines, and further, since the evolutionary ecology of plant defense to herbivory has been extensively examined in this species (Tiffin 2000; Tiffin and Rausher 1999), but the genetic factors underlying these defenses have yet to be investigated. We identified 289 transcripts that belonged to one of 34 families (121 subfamilies) of cytochrome P450 genes, and these genes were predominantly sequenced at near full-length. The largest superfamily of P450 genes in the *I. purpurea* transcriptome is the CYP82 clan, members of which mediate plant-specific alkaloid pathways (Nelson and Werck-Reichhart 2011; Nelson *et al.* 2004) and are induced in response to wounding and cold stress (Whitbred and Schuler 2000). Another large family found in the *I. purpurea* transcriptome with 24 transcripts is the CYP736 clan—this group has been indicated in defense response to microbial infection in grapevine (Cheng *et al.* 2010). The transcript that we identified as overexpressed in an RNA-seq survey belongs to the CYP82 clan, and the C22 subfamily. Very little information is currently available for this specific subfamily beyond that of being involved in an oxidation-reduction process (GO category, GO: 0055114). However, it is interesting that the most abundant families of cytochrome P450 genes within the *I. purpurea* transcriptome are involved in pathways that mediate responses to environmental stress.

### Gene expression differences of artificially diverged herbicide-resistant selection lines

We uncovered 19 differentially expressed genes, seven of which were up-regulated and 12 that were down-regulated in resistant lines compared with susceptible lines. As previously mentioned, a cytochrome P450 transcript was up-regulated in the resistant lines and is of special interest since cytochrome P450 genes are indicated in xenobiotic detoxification in plants and other organisms (Gueguen *et al.* 2006). Of the other transcripts up-regulated in the R lines, two could be directly linked to signaling, one of which, a “serine/threonine-protein kinase AtPK7-like” gene, is a member of a diverse group of proteins associated with growth signaling pathways (Bogre *et al.* 2003). Three of the other seven up-regulated genes—two (-)-germacrene D-synthases and a pto-like serine/threonine kinase—are related to defense. Germacrene D-synthase is associated with insect and microbe defense in plant species (Arimura *et al.* 2000), and *Pto* is related to microbial resistance in tobacco (Thilmony *et al.* 1995). The increased expression of the antimicrobial genes in resistant lines is especially interesting, since the “secondary mode of action” of glyphosate is to increase a range of diseases in crop species (Johal and Huber 2009) and predisposition to disease has been hypothesized to underlie glyphosate’s efficacy (Johal and Huber 2009). Thus, our lines may be resistant to changes to the microbial community and/or to microbial infection after glyphosate application. Interestingly, we did not detect increased expression of EPSP synthase in our resistant lines, suggesting that resistance, in *I. purpurea*, is not conferred by overexpression of this protein, as it is in other glyphosate resistant species.

Of the 12 transcripts that were down-regulated in resistant lines, 9 resembled receptor-like kinases, a class of *trans*-membrane proteins that are primarily involved with cellular signaling (Stone and Walker 1995). A member of the ceramidase protein family was also down-regulated in resistant lines compared with susceptible lines—this gene is involved in cell growth arrest, differentiation, cell senescence, cell migration, and cell-adhesion processes (Mao and Obeid 2008). The final two genes that were down-regulated in resistant lines were a pectin methylesterase and vicianin hydrolase-like gene. Pectin methylesterase is associated with development and stiffening of the cell wall and is also suggested to play a role in stress response (Pelloux *et al.* 2007). Vicianin hydrolase is a member of the glycoside hydrolase protein family, which is responsible for breaking down polysaccharides (Ahn *et al.* 2007). Thus, resistant individuals down-regulate gene expression of signaling-associated loci relative to susceptible individuals—specifically loci that are indicated in cell senescence, cell wall development, and polysaccharide breakdown.

Although this large-scale transcriptomic survey was performed primarily to determine whether there were detectable, early gene expression changes between selection lines following challenge with the herbicide, we now have candidate loci that may be involved in the resistance mechanism. For example, the up-regulation of cytochrome P450 in the resistant lines could indicate that glyphosate resistance is conferred through detoxification and metabolism in the common morning glory. Another interesting potential mechanism of resistance in this species that has previously been suggested (Sammons and Gaines 2014) is an altered pattern of glyphosate translocation throughout the plant. Previous work has shown that glyphosate enters companion cells within morning glory leaves, but then does not move into the transpiration stream via the phloem as in other species (Gaines *et al.* 2010). Here, we find that resistant plants down-regulate a ceramidase protein family transcript and a pectin methylesterase—both of which are localized to the plasmodesmata, or microscopic channels through which sugars and other nutrients move between plant cells. The down-regulation of transcripts that are involved with transport in resistant lines—and specifically transcripts that may be responsible for the transport of glyphosate to the apical meristems—provides another line of evidence that resistance in this species could be due to altered translocation.

In addition, it is important to note that we assessed gene expression changes of artificially selected glyphosate resistant and susceptible lines after exposure to the herbicide—we did not present data on plants that had not been challenged with the herbicide. Preliminary analyses of the pectin methylesterase and vicianin hydrolase-like gene using a qPCR assay of exposed and nonexposed replicate individuals indicate that the gene expression changes are not induced by the herbicide in either R or S lines (data not shown); however, cytochrome P450 genes often are induced by stressors, and, as such, we are actively pursuing the potential that the P450 genes are differentially induced in the artificially selected R and S lines. This is especially relevant in light of preliminary evidence indicating a trade-off associated with resistance in this species.

Despite the interesting mechanistic possibilities that the gene expression survey presented here suggests, several very important caveats should be considered before attributing any causal relationship between the differentially expressed genes and resistance. First, large differences in gene expression do not identify the heritable basis of the trait being investigated—such a question is best addressed with a mapping or candidate gene approach, both of which are currently in progress. Second, the trait may be influenced by subtle differences in gene expression that will not be statistically significant in the RNA-seq analysis. Third, RNA-seq provides no insight for potentially mechanistic contributions of upstream promoter and intronic regions, and finally, RNA-seq analysis tends to assume that the relative abundance of mRNA results in equal relative abundances of protein and function, and this is not always a safe assumption (Greenbaum *et al.* 2003). QTL mapping followed by functional verification of candidate loci will be necessary to identify the causal genetic component underlying resistance in this species.

*I. purpurea* has been a model plant in evolutionary ecology and genetics investigations since the early 1990s—in this body of work, researchers have used quantitative genetics designs, assays of variation within single genes, and basic field ecology experiments to examine the genetic factors and evolutionary forces that underlie mixed mating systems, floral color polymorphisms, plant-herbivore and plant-parasite interactions, as well as herbicide resistance and tolerance (Baucom *et al.* 2011). Genomics tools have been lacking in this species, however, and the annotated transcriptome presented here will aid future investigations of these topics in this species and other closely related *Ipomoea* species. This work also provides a valuable resource to researchers interested in utilizing the close relationship between the Convolvulaceae and the Solanaceae for phylogenetic and comparative genomics examinations. As an example of the utility of the annotated transcriptome, here we assessed gene expression changes associated with glyphosate resistance—notably, we did not detect increased expression of EPSP synthase in the resistant lines compared to susceptible lines. This result indicates resistance in *I. purpurea* is not conferred by overproduction of this enzyme as it is in other glyphosate resistant weedy plants, but rather, is likely due to changes in nontarget loci.

## Supplementary Material

Supporting Information
