# Supplementary material for: De Novo Assembly and Annotation of the Transcriptome of the Agricultural Weed Ipomoea purpurea Uncovers Gene Expression Changes Associated with Herbicide Resistance
Source: G3 (Bethesda). 2014 Aug 25;4(10):2035–47. doi: 10.1534/g3.114.013508 (PMC4199709; doi:10.1534/g3.114.013508)
Supplement: Supporting Information [file supp_4_10_2035__index.html]

De Novo Assembly and Annotation of the Transcriptome of the Agricultural Weed Ipomoea purpurea Uncovers Gene Expression Changes Associated with Herbicide Resistance — Supporting Information 

# *De Novo* Assembly and Annotation of the Transcriptome of the Agricultural Weed *Ipomoea purpurea* Uncovers Gene Expression Changes Associated with Herbicide Resistance

## Supporting Information for Leslie and Baucom *et al.*, 2014

**Files in this Data Supplement:**

- Supporting Information - File S1, Figures S1-S5, and Tables S1-S6 (PDF, 814 KB)
- File S1 - Supplemental methods, results, tables and figures. (PDF, 118 KB)
- Figure S1 - Mapping specificity in RNA-Seq expression profiles. (PDF, 469 KB)
- Figure S2 - Binned edgeR counts per million plotted against binned scaffold lengths. (PDF, 219 KB)
- Figure S3 - Average expression (FPKM) values according to transcript length shown by (A) transcripts that were annotated by blastx to the NCBI nr database, and (B) transcripts that could not be annotated by blast. (PDF, 161 KB)
- Figure S4 - The distribution of transcripts within the *I. purpurea* transcriptome assigned to GOSlim categories within the Biological Process, Molecular Function and Cellular Component GO categories. (PDF, 190 KB)
- Figure S5 - Examination of the protein length of cytochrome p450 transcriptoms from the *I. purpurea* transcriptome as in Figure 3. (PDF, 122 KB)
- Table S2 - RNA-Seq analysis of common morning glory (*I. purpurea*) using the Illumina hiseq2000L sequencing yields and quality filtering. (PDF, 111 KB)
- Table S3 - Efficiency of mapping Illumina hiseq2000 RNA-Seq reads against the *I. purpurea* reference transcriptome, a de novo cDNA assembly. (PDF, 112 KB)
- Table S4 - Primers developed for the qPCR verification of DEGs. (PDF, 86 KB)
- Table S5 - Differentially expressed genes as identified by edgeR after binning scaffolds based on length (bins were defined as 200-1000 bp, 1001-2000 bp, 2001-3000 bp, and >3000 bp). (PDF, 101 KB)
- Table S6 - Annotation of the cytochrome P450 genes in the *Ipomoea* transcriptome. (.xlsx, 39 KB)
- Table S1 - Annotation of the *I. purpurea* reference transcriptome. (.zip, 17 MB)
