# Supplementary material for: De Novo Assembly and Annotation of the Transcriptome of the Agricultural Weed Ipomoea purpurea Uncovers Gene Expression Changes Associated with Herbicide Resistance
Source: G3 (Bethesda). 2014 Aug 25;4(10):2035–47. doi: 10.1534/g3.114.013508 (PMC4199709; doi:10.1534/g3.114.013508)
Supplement: Supporting Information [file supp_g3.114.013508_013508SI.pdf]

G3: Genes | Genomes | Genetics

De novo assembly and annotation of the transcriptome of the agricultural weed *Ipomoea purpurea* uncovers gene expression changes associated with herbicide resistance

Trent Leslie[1]
Regina S Baucom[2]

[1]Department of Biological Sciences
University of Cincinnati
Cincinnati, OH 45221
trentleslie@gmail.com

[2]2059 Kraus Natural Science Building
Department of Ecology and Evolutionary Biology
University of Michigan
Ann Arbor, MI 48103
rsbaucom@umich.edu
(734) 647-8490 (office phone)
(734) 763-0544 (fax)
[2]author for correspondence

Data Archival Location
-RNAseq data is available in the SRA database of NCBI, and can be accessed via this link:
http://www.ncbi.nlm.nih.gov/bioproject/?term=PRJNA216984

-The *Ipomoea* transcriptome is available through the 1kp plant transcriptome project and can be accessed through http://www.onekp.com/

-Annotation of the *I. purpurea* reference transcriptome is made available as supplemental material and can be found in TableS1.txt

DOI: 10.1534/g3.114.013508

File S1

Supplemental methods, results, tables and figures

Methods—Mapping specificity and bias

We examined both the position on the scaffold to which the reads mapped and the scaffold length to determine if they might influence our ability to assess differences in gene expression between resistant and susceptible lines. The median mapping position of the reads across all scaffolds was base pair 616; nearly one percent of all reads mapped to the first base pair of the scaffold (0.8%, Figure S2b). We found a weak positive correlation between the mean mapping position of all the reads on a scaffold and the number of reads that mapped to the scaffolds ($R^2 = 0.28$, $p < 2.2e\text{-}16$, Figure S2c). This suggests that scaffolds with average mapping positions farther from the 3' end of the scaffold exhibit higher expression than those with average mapping positions closer to the 3' end. Likewise, a moderate positive correlation exists between the edgeR counts per million across all samples and the scaffold length ($R^2 = 0.30$, $p < 2.2e\text{-}16$, Figure S2d), suggesting that longer scaffolds may exhibit higher expression than shorter scaffolds. These results should not impact our broad comparison of gene expression differences, as the same genes and thus scaffolds are being compared between R and S individuals, and, the correlations between variables are relatively modest. However, if a short scaffold is the causal agent(s) underlying resistance, and the average expression of short scaffolds is lower than longer scaffolds, then scaffold length bias in our data could increase our probability of type II error. To test for such an effect, we performed an analysis wherein scaffolds were binned according to length (200-1,000 bp, 1,001-2,000 bp, 2,001-3,000 bp, and >3,000 bp) and gene expression patterns were assessed between R and S individuals as for the entire dataset.

Results—Mapping specificity and bias

When scaffolds were binned according to length, edgeR identified 17 differentially expressed genes, 15 of which were identified in the analysis of the full dataset (Figures S3; Table S4). Four genes—all within the 200-1000 bp bin – were lost compared to the overall dataset, and two genes – both from the 1001-2000 bp bin – were gained. Thus, we do not find that the relationship between read count and scaffold length significantly influences our ability to uncover differential expression of the shorter scaffolds. The two genes that were gained by binning were annotated by Blast2GO as an ATP-binding protein (*XP_002518555*) and indoleacetic acid-induced-like protein (*XP_002264963*).

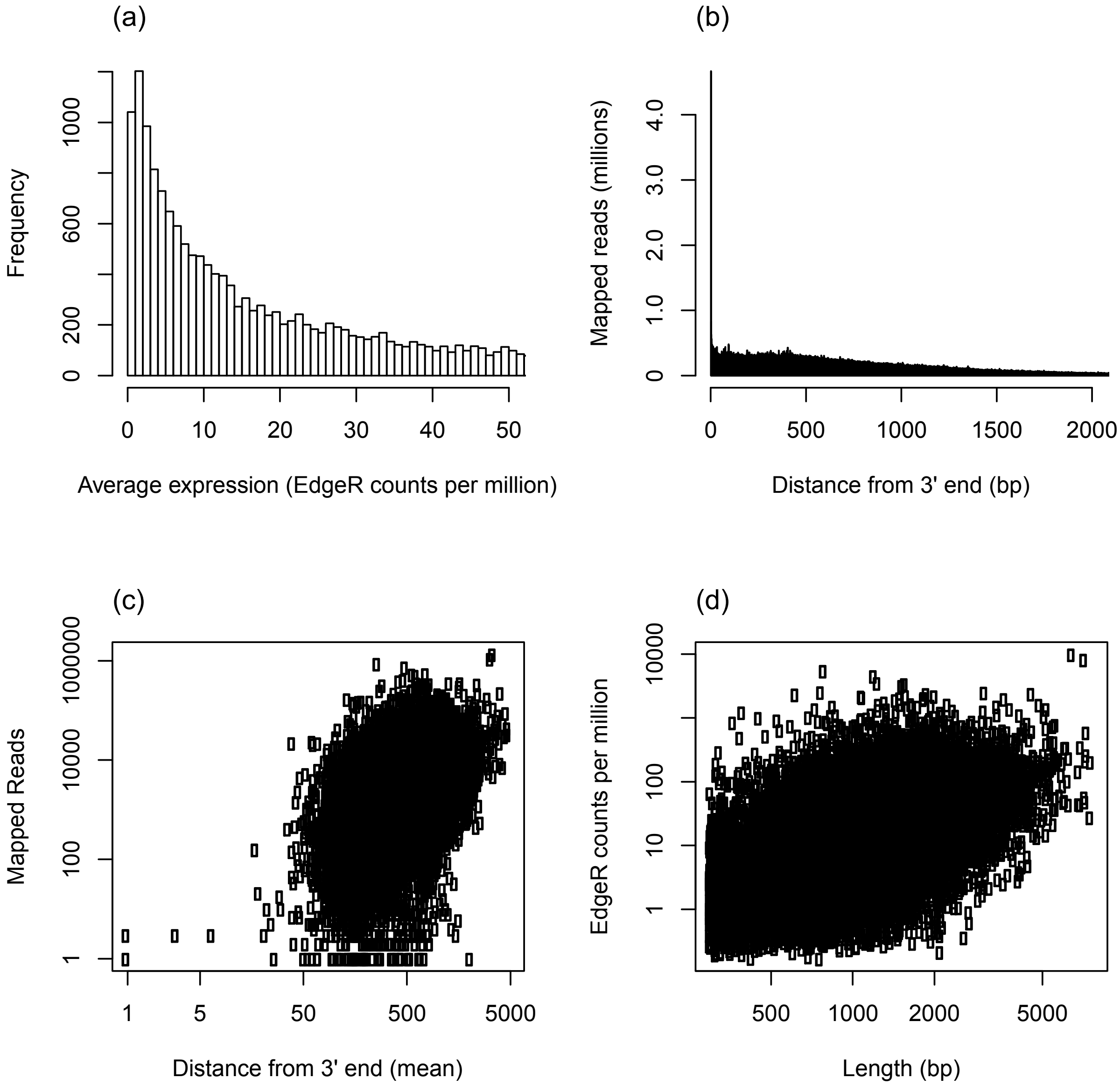

Figure S1 Mapping specificity in RNA-Seq expression profiles. Histograms of (a) average expression (edgeR counts per million) and (b) read mapping positions across samples. (c) Number of mapped reads vs. mean mapping position ($R^2 = 0.28$, $p < 2.2e\text{-}16$). This illustrates the relationship between the number of reads that map to a particular scaffold and the mean mapping position of those reads. (d) The relationship between edgeR counts per million and scaffold length ($R = 0.30$, $p < 2.2e\text{-}16$).

Table S1 Annotation of the *I. purpurea* reference transcriptome.

Available for download as a .zip file at http://www.g3journal.org/lookup/suppl/doi:10.1534/g3.114.013508/-/DC1

Table S2 RNA-Seq analysis of common morning glory (*I. purpurea*) using the Illumina hiseq2000L sequencing yields and quality filtering.

Treatments	Sample	Reads (M)	HQ (M)	HQ (%)
Glyphosate Susceptible	068-1-1	65.8	62.9	95.6
	077-1-2	67.4	64.4	95.5
	079-1-3	41.2	39.0	94.7
Glyphosate Resistant	336-1-2	152.0	146.9	96.6
	351-1-2	57.5	54.2	94.3
	358-1-3	78.3	75.0	95.8
Total raw reads (millions)		462.2	442.4	
Average per sample		77.0	73.7	95.4

Table S3 Efficiency of mapping Illumina hiseq2000 RNA-Seq reads against the *I. purpurea* reference transcriptome, a de novo cDNA assembly. Note Bowtie2 reports on paired reads, effectively halving the absolute raw and HQ read counts seen in Table S1.

Treatments	Sample	Mapped Reads (M)	Mapped Reads (%)	Unique Alignments (M)	Unique Alignments (%)
Glyphosate Susceptible	068-1-1	14.9	47.4	12.8	40.6
	077-1-2	15.1	46.8	12.8	39.6
	079-1-3	9.1	46.5	7.6	39.1
Glyphosate Resistant	336-1-2	33.5	45.6	28.7	39.1
	351-1-2	14.6	65.7	13.6	54.7
	358-1-3	22.2	59.1	19.0	50.6
Total mapped reads (millions)		109.4		94.5	
Percent of HQ reads mapped		49.4		42.8	
Percent of raw reads mapped		47.4		40.8	

Table S4 Primers developed for the qPCR verification of DEGs.

Sequence Name	Sequence	Tm (50mM NaCl) C
abc-b_2016948_178F	CTT TGC TGG CTT TCT TGG AC	54.52741302
abc-b_2016948_178R	GGT GAT CGA ATG GCG TTA CT	54.99775102
atpbin-2010370_162F	CTC CGC TCT TTC TTC CAA TG	53.5183376
atpbin-2010370_162R	TGC AGT ATA TCG GTG GTG GA	55.53902069
brass-2061274_150F	CTG GTA TAA CGA GCC GGT GT	56.76005614
brass-2061274_150R	GCA TAG ATT TCG ACG GCA TT	53.33061546
ceram-2056577_177F	GAG CCA GGC TTG AGA GTG TT	57.31439485
ceram-2056577_177R	GCT GTT TGC AAT GTG AGC AT	54.56376496
cysrec-2011172_162F	GGT TCC CTA GCT CCC TCA TC	56.77992894
cysrec-2011172_162R	ACT AGG TCA CCG CCT CTT CA	57.77335135
germd-2001731_176F	TTG AGC CAA ATG GAA CAA CA	52.8778193
germd-2001731_176R	CCA AAA GTA GCC TTC CAC CA	55.03216815
glut-s_2002932_175F	TTT TGT GCA CTT GGG TTG AA	53.31376738
glut-s_2002932_175R	GCA CCA GTT TCA ATT GGC TT	54.2963209
helica-2013762_163F	TTG CAA CTG GCT TTC AAC AG	54.15438015
helica-2013762_163R	ATT TTC TGC AAA CCT GGT GG	53.78453676
P450_1-2005659_173F	TGT ATC AAC CAC GGT CTC CA	55.65938391
P450_1-2005659_173R	CGC GCT TTC CTA TCT ACC AG	55.29411425
P450_2-2003522_172F	GAG CAA AAA CCT TGC AGA CC	54.76344105
P450_2-2003522_172R	AAT TGC TGG ACA CCA ACC TC	55.56952121
P450a_2003581_168F	AGT GCT GGT GGT TAG CGA CT	58.78593189
P450a_2003581_168R	ATA AGT TTG CGA ATC CCA CG	53.24697226
pecmet_2017152_161F	TAG AAT TGC CGC TGA CTG TG	55.20539275
pecmet_2017152_161R	GAT GGG TTC ATA GCC CAA GA	54.40150801
proto_2011804_157F	CGG AGT TCT GGT ATT GGG GT	56.64581809
proto_2011804_157R	CAT AAA GGT GGC GAC GAT GG	56.10508724

serthre-2055046_161F	GGA GAA GGG AAG TCT CGA CC	56.78871641
serthre-2055046_161R	TGA TCG GAG TGT CCA ATG AG	54.20362614
vichyd_2063945_178F	TAT TTG GTG ATC GCG TGA AA	52.35251201
vichyd_2063945_178R	TGA GCA AGA AGC AAA TGG TG	53.72764614
wb-abc_2017606_160F	AAG CTT CTG TTC CTG GAC GA	56.15266566
wb-abc_2017606_160R	GGC ACA GGC TAG TGA AGA GG	57.64456754

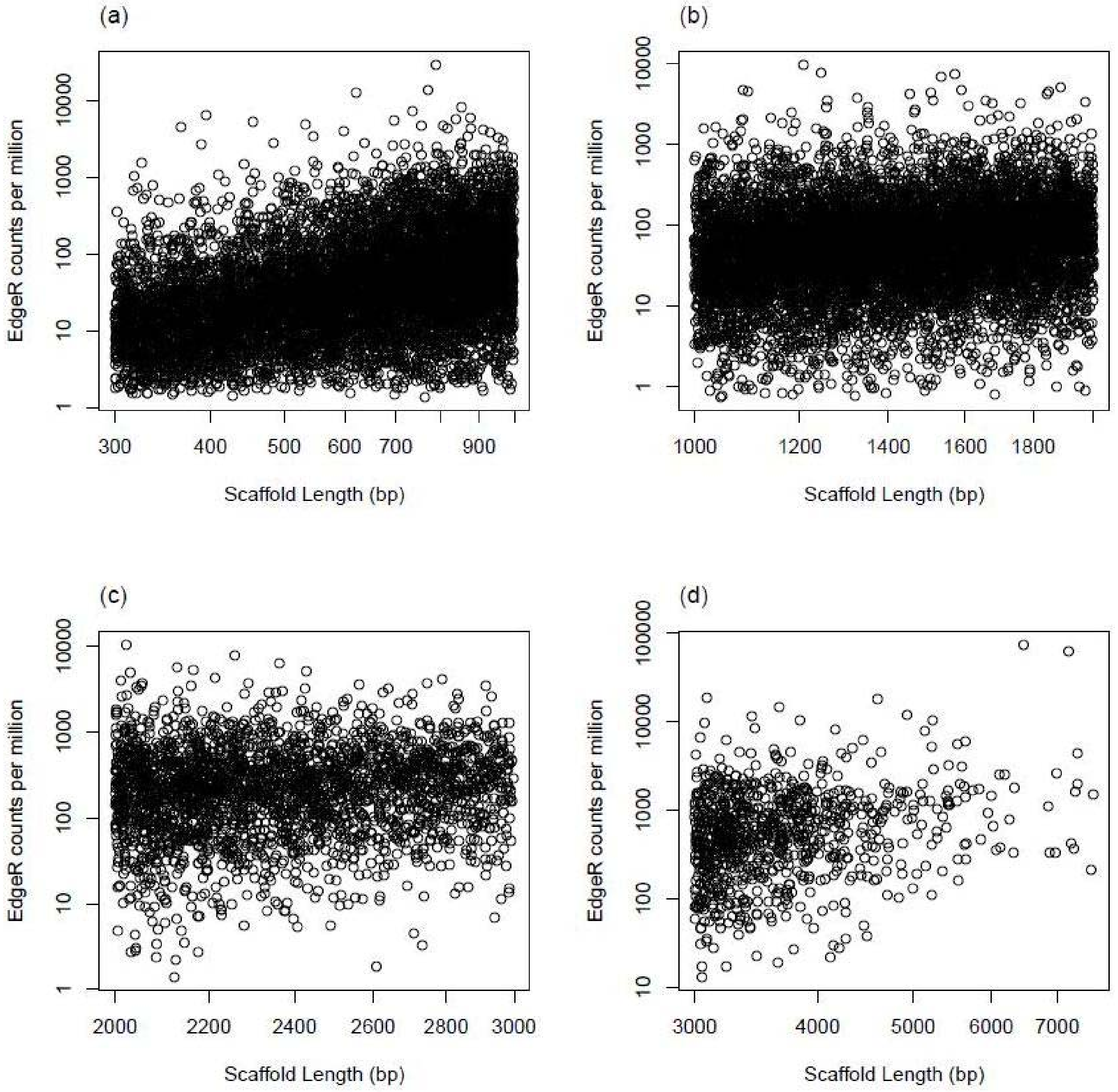

Figure S2 Binned edgeR counts per million plotted against binned scaffold lengths. (a) 200-1000 bp scaffolds ($p < 2.2e-16$, R^2=0.159) (b) 1001-2000 bp scaffolds ($p < 2.2e-16$, R^2=0.02655) (c) 2001-3000 bp scaffolds ($p = 5.67e-7$, R^2=0.00925) (d) >3000 bp scaffolds (p=9.57e-12, R^2=0.0504)

Table S5 Differentially expressed genes as identified by edgeR after binning scaffolds based on length (bins were defined as 200-1000 bp, 1001-2000 bp, 2001-3000 bp, and >3000 bp). Note resistant/susceptible log2 fold changes.

rank	bin (bp)	Scaffold ID	topblast	logFC	logCPM	PValue	padj
1	1,001-2,000	2009240	protein	-4.956514395	4.417191331	1.60E-13	1.33E-09
2	1,001-2,000	2063945	vicianin hydrolase-like	-2.951166513	5.431831016	8.98E-10	3.72E-06
3	1,001-2,000	2001731	(-)-germacrene d synthase	4.77309533	3.683966828	7.52E-09	2.07E-05
4	200-1,000	2056577	ceramidase family protein	-7.490398504	3.019096973	5.90E-09	4.79E-05
5	1,001-2,000	2009241	protein	-4.183410284	3.563076107	4.15E-08	8.60E-05
6	1,001-2,000	2005233	(-)-germacrene d synthase	5.375616041	2.268376148	9.28E-08	0.000153718
7	1,001-2,000	2010370	atp binding	-4.830721823	4.01726904	1.23E-06	0.001701055
8	1,001-2,000	2002437	protein	2.476840484	5.144280666	3.69E-06	0.004362936
9	1,001-2,000	2061274	brassinosteroid insensitive 1-associated receptor kinase 1	-4.079306873	4.148482421	4.98E-06	0.005147925
10	200-1,000	2003581	cytochrome p450 82a3-like	3.295661347	3.632279277	2.64E-06	0.010717398
11	1,001-2,000	2004377	protein	-3.861554692	3.112662587	3.24E-05	0.029797255
12	1,001-2,000	2013762	u5 small nuclear ribonucleoprotein helicase	5.089691419	1.196586827	3.61E-05	0.02985129
13	1,001-2,000	2009597	atp binding	-1.930162869	8.43404822	4.09E-05	0.030796464
14	1,001-2,000	2017152	pectin methylesterase	-3.254112192	4.114218356	4.61E-05	0.031807096
15	200-1,000	2054556	protein	3.437708896	2.968028123	1.75E-05	0.036093279
16	200-1,000	2059855	protein kinase	4.123441785	4.96632482	1.78E-05	0.036093279
17	1,001-2,000	2010065	indoleacetic acid-induced-like protein	-2.027993775	4.890619041	5.67E-05	0.036114335

Table S6   Annotation of the cytochrome P450 genes in the *Ipomoea* transcriptome.

Available for download as an Excel file at http://www.g3journal.org/lookup/suppl/doi:10.1534/g3.114.013508/-/DC1

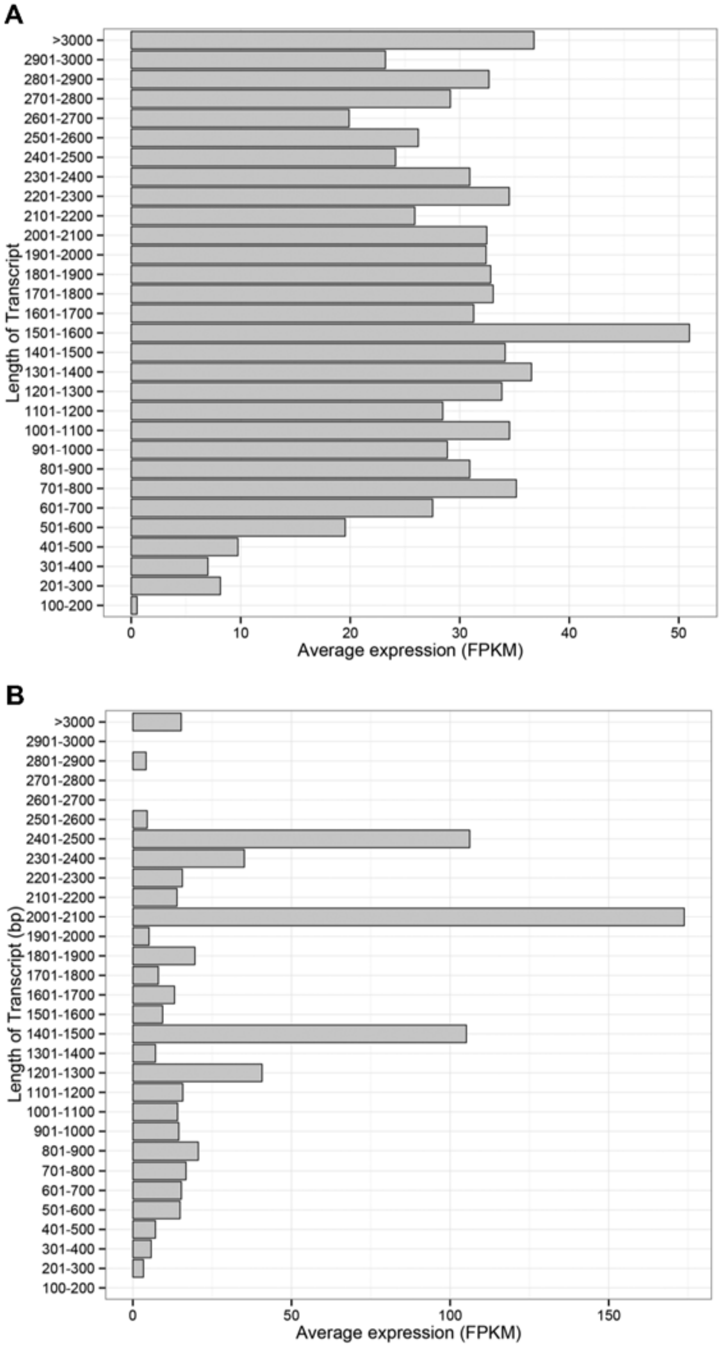

Figure S3 Average expression (FPKM) values according to transcript length shown by (A) transcripts that were annotated by blastx to the NCBI nr database, and (B) transcripts that could not be annotated by blast.

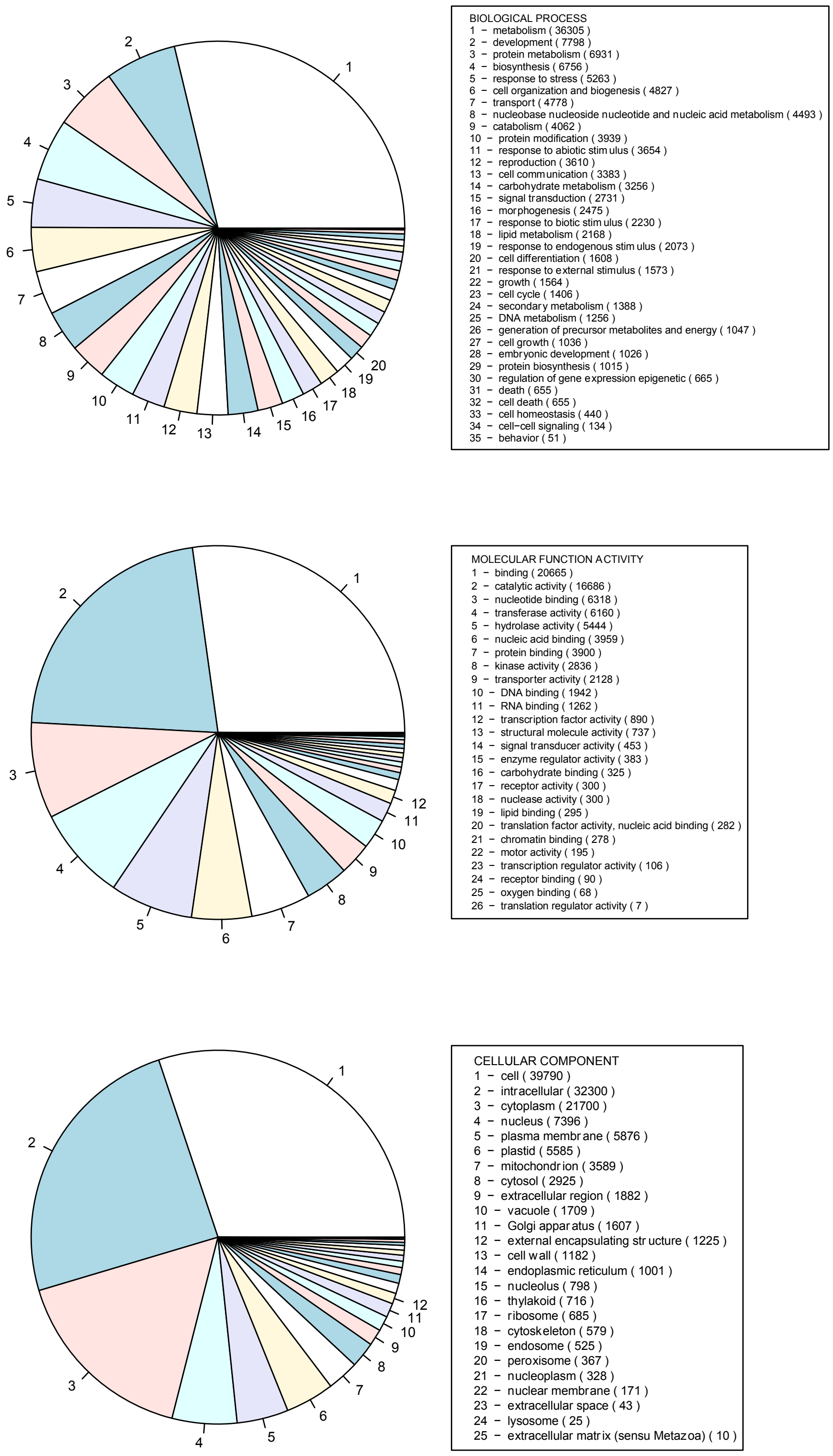

Figure S4 The distribution of transcripts within the *I. purpurea* transcriptome assigned to GOSlim categories within the Biological Process, Molecular Function and Cellular Component GO categories.

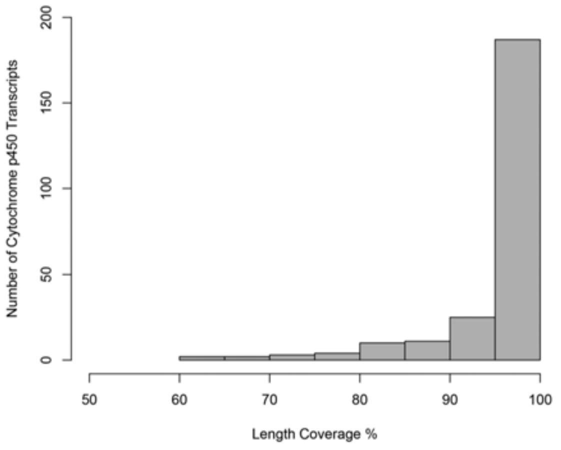

Figure S5 Examination of the protein length of cytochrome p450 transcriptoms from the *I. purpurea* transcriptome as in Figure 3.
