# Supplementary material for: De Novo Assembly and Annotation of the Transcriptome of the Agricultural Weed Ipomoea purpurea Uncovers Gene Expression Changes Associated with Herbicide Resistance
Source: G3 (Bethesda). 2014 Aug 25;4(10):2035–47. doi: 10.1534/g3.114.013508 (PMC4199709; doi:10.1534/g3.114.013508)
Supplement: Supporting Information [file supp_g3.114.013508_FileS1.pdf]

File S1

Supplemental methods, results, tables and figures

Methods—Mapping specificity and bias

We examined both the position on the scaffold to which the reads mapped and the scaffold length to determine if they might influence our ability to assess differences in gene expression between resistant and susceptible lines. The median mapping position of the reads across all scaffolds was base pair 616; nearly one percent of all reads mapped to the first base pair of the scaffold (0.8%, Figure S2b). We found a weak positive correlation between the mean mapping position of all the reads on a scaffold and the number of reads that mapped to the scaffolds ($R^2 = 0.28$, $p < 2.2e-16$, Figure S2c). This suggests that scaffolds with average mapping positions farther from the 3' end of the scaffold exhibit higher expression than those with average mapping positions closer to the 3' end. Likewise, a moderate positive correlation exists between the edgeR counts per million across all samples and the scaffold length ($R^2 = 0.30$, $p < 2.2e-16$, Figure S2d), suggesting that longer scaffolds may exhibit higher expression than shorter scaffolds. These results should not impact our broad comparison of gene expression differences, as the same genes and thus scaffolds are being compared between R and S individuals, and, the correlations between variables are relatively modest. However, if a short scaffold is the causal agent(s) underlying resistance, and the average expression of short scaffolds is lower than longer scaffolds, then scaffold length bias in our data could increase our probability of type II error. To test for such an effect, we performed an analysis wherein scaffolds were binned according to length (200-1,000 bp, 1,001-2,000 bp, 2,001-3,000 bp, and >3,000 bp) and gene expression patterns were assessed between R and S individuals as for the entire dataset.

Results—Mapping specificity and bias

When scaffolds were binned according to length, edgeR identified 17 differentially expressed genes, 15 of which were identified in the analysis of the full dataset (Figures S3; Table S4). Four genes—all within the 200-1000 bp bin – were lost compared to the overall dataset, and two genes – both from the 1001-2000 bp bin – were gained. Thus, we do not find that the relationship between read count and scaffold length significantly influences our ability to uncover differential expression of the shorter scaffolds. The two genes that were gained by binning were annotated by Blast2GO as an ATP-binding protein (*XP_002518555*) and indoleacetic acid-induced-like protein (*XP_002264963*).
