# Supplementary material for: De Novo Assembly and Annotation of the Transcriptome of the Agricultural Weed Ipomoea purpurea Uncovers Gene Expression Changes Associated with Herbicide Resistance
Source: G3 (Bethesda). 2014 Aug 25;4(10):2035–47. doi: 10.1534/g3.114.013508 (PMC4199709; doi:10.1534/g3.114.013508)
Supplement: Supporting Information [file supp_g3.114.013508_FigureS1.pdf]

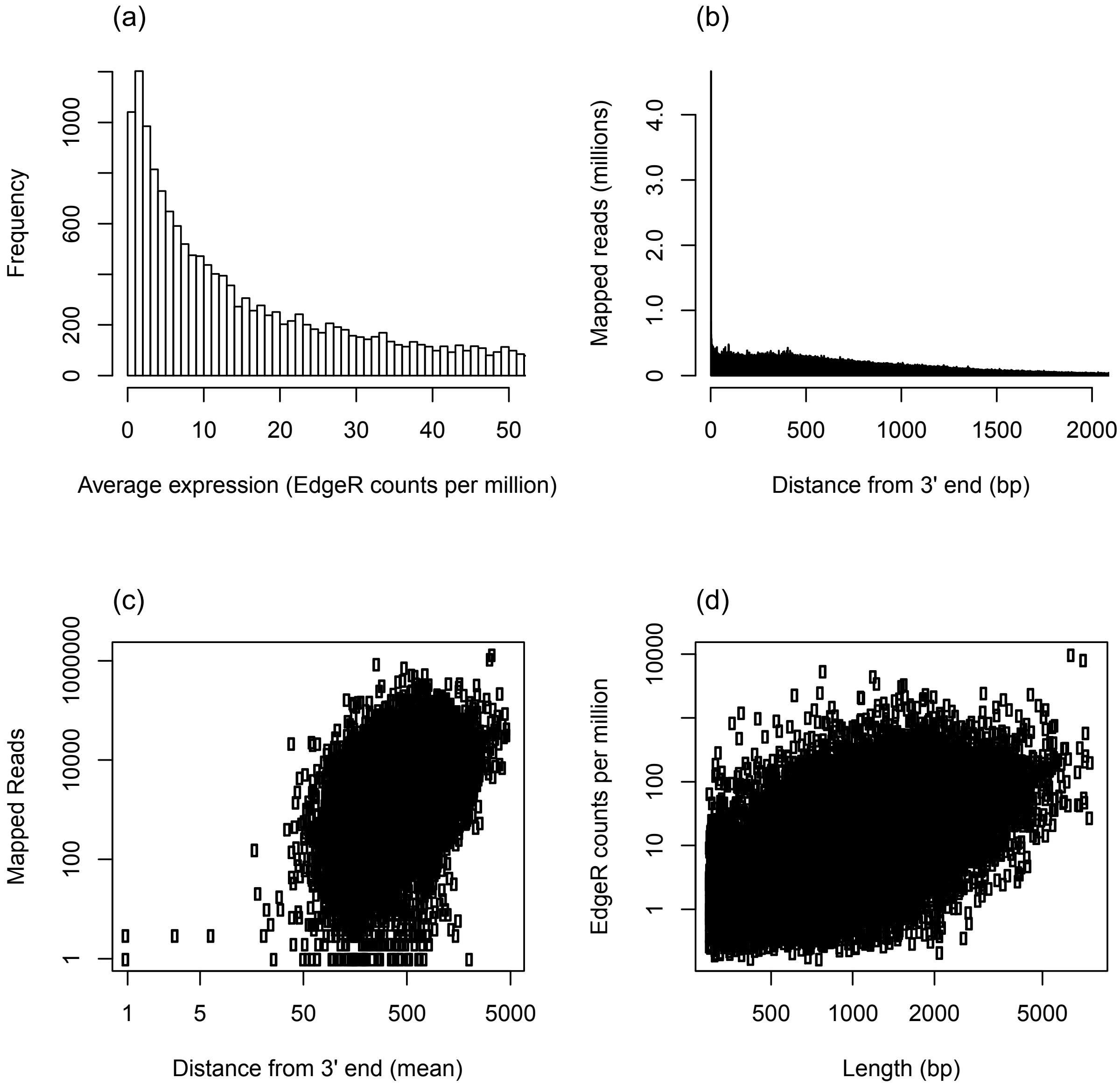

Figure S1 Mapping specificity in RNA-Seq expression profiles. Histograms of (a) average expression (edgeR counts per million) and (b) read mapping positions across samples. (c) Number of mapped reads vs. mean mapping position ($R^2 = 0.28$, $p < 2.2e\text{-}16$). This illustrates the relationship between the number of reads that map to a particular scaffold and the mean mapping position of those reads. (d) The relationship between edgeR counts per million and scaffold length ($R = 0.30$, $p < 2.2e\text{-}16$).
