# Supplementary material for: De Novo Assembly and Annotation of the Transcriptome of the Agricultural Weed Ipomoea purpurea Uncovers Gene Expression Changes Associated with Herbicide Resistance
Source: G3 (Bethesda). 2014 Aug 25;4(10):2035–47. doi: 10.1534/g3.114.013508 (PMC4199709; doi:10.1534/g3.114.013508)
Supplement: Supporting Information [file supp_g3.114.013508_FigureS2.pdf]

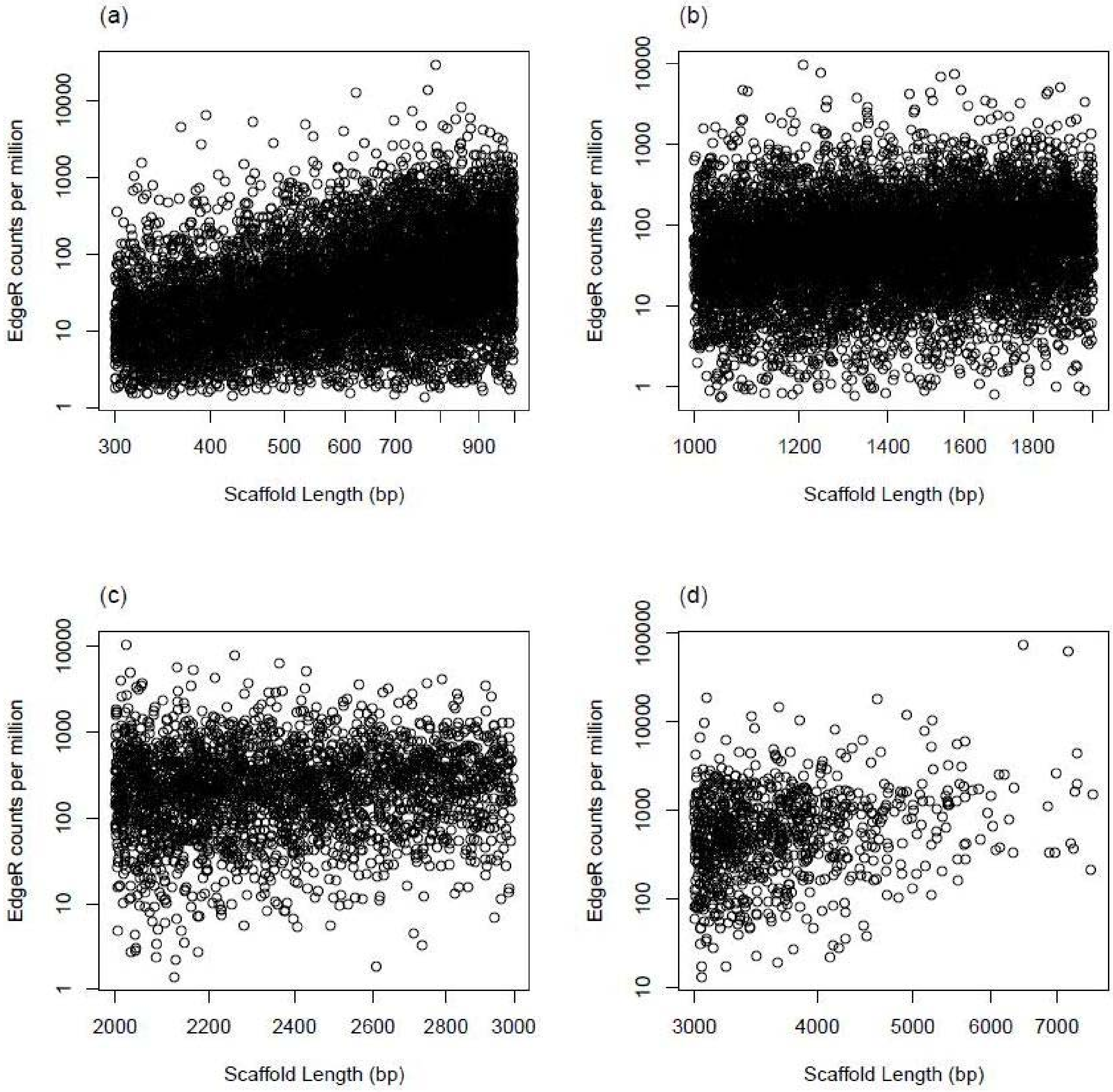

Figure S2 Binned edgeR counts per million plotted against binned scaffold lengths. (a) 200-1000 bp scaffolds ($p < 2.2e-16$, $R^2=0.159$) (b) 1001-2000 bp scaffolds ($p < 2.2e-16$, $R^2=0.02655$) (c) 2001-3000 bp scaffolds ($p = 5.67e-7$, $R^2=0.00925$) (d) >3000 bp scaffolds ($p=9.57e-12$, $R^2=0.0504$)
