# Supplementary material for: De Novo Assembly and Annotation of the Transcriptome of the Agricultural Weed Ipomoea purpurea Uncovers Gene Expression Changes Associated with Herbicide Resistance
Source: G3 (Bethesda). 2014 Aug 25;4(10):2035–47. doi: 10.1534/g3.114.013508 (PMC4199709; doi:10.1534/g3.114.013508)
Supplement: Supporting Information [file supp_g3.114.013508_FigureS3.pdf]

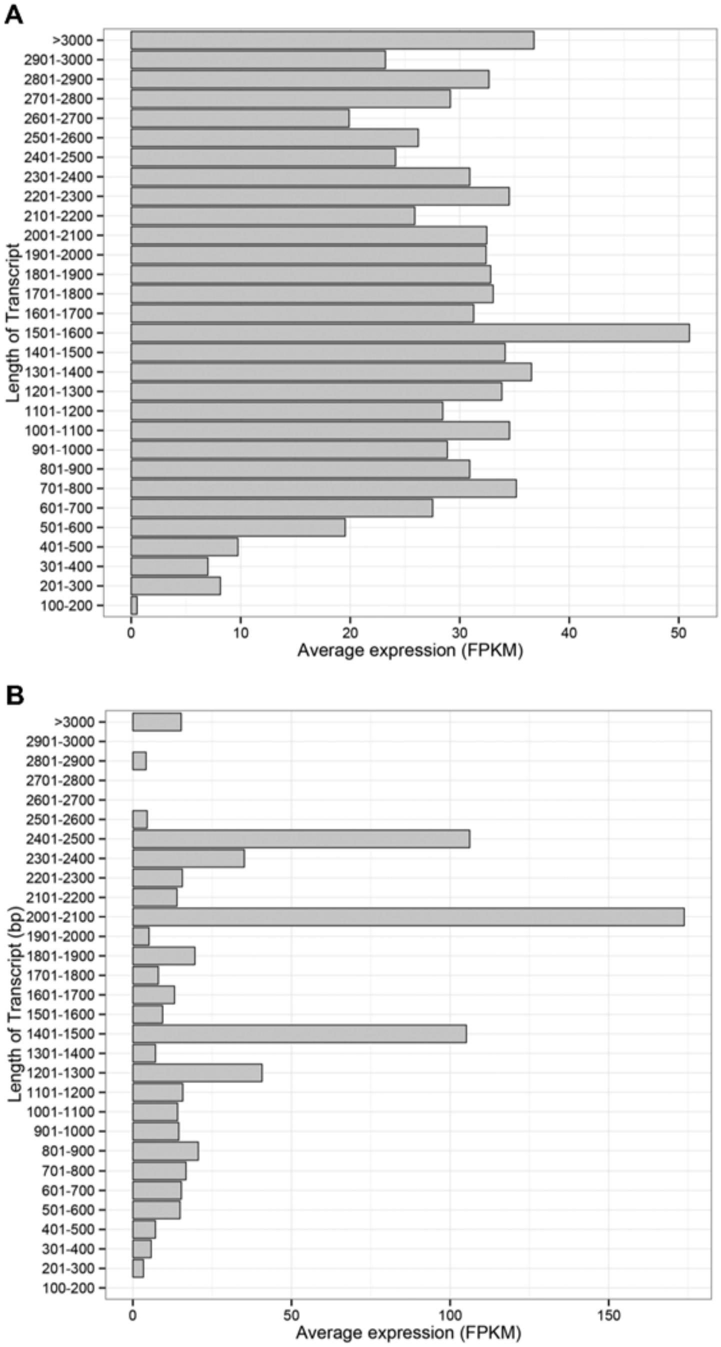

Figure S3 Average expression (FPKM) values according to transcript length shown by (A) transcripts that were annotated by blastx to the NCBI nr database, and (B) transcripts that could not be annotated by blast.
