# Supplementary material for: De Novo Assembly and Annotation of the Transcriptome of the Agricultural Weed Ipomoea purpurea Uncovers Gene Expression Changes Associated with Herbicide Resistance
Source: G3 (Bethesda). 2014 Aug 25;4(10):2035–47. doi: 10.1534/g3.114.013508 (PMC4199709; doi:10.1534/g3.114.013508)
Supplement: Supporting Information [file supp_g3.114.013508_FigureS4.pdf]

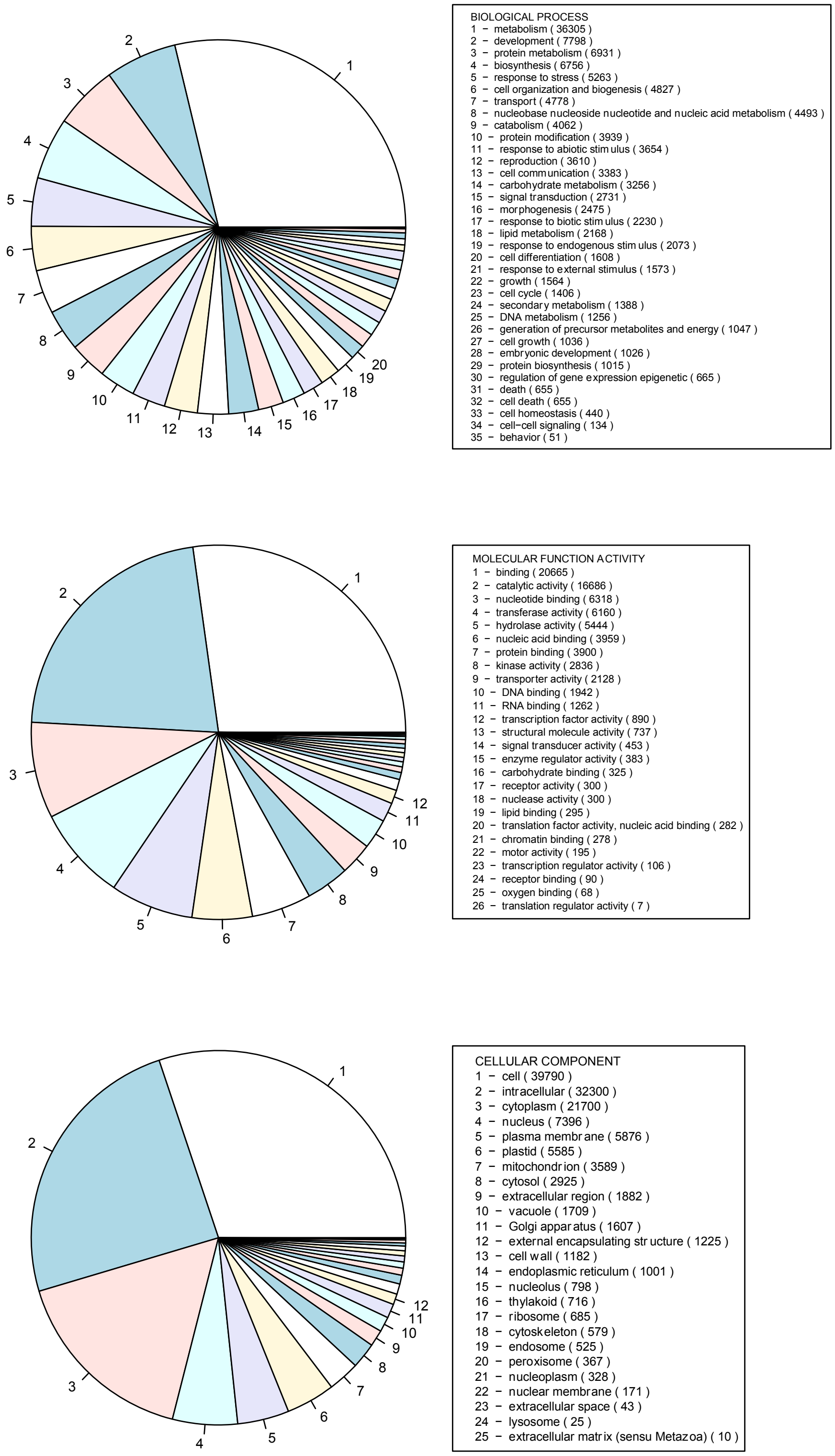

Figure S4 The distribution of transcripts within the *I. purpurea* transcriptome assigned to GOSlim categories within the Biological Process, Molecular Function and Cellular Component GO categories.
