# Supplementary material for: De Novo Assembly and Annotation of the Transcriptome of the Agricultural Weed Ipomoea purpurea Uncovers Gene Expression Changes Associated with Herbicide Resistance
Source: G3 (Bethesda). 2014 Aug 25;4(10):2035–47. doi: 10.1534/g3.114.013508 (PMC4199709; doi:10.1534/g3.114.013508)
Supplement: Supporting Information [file supp_g3.114.013508_TableS2.pdf]

Table S2 RNA-Seq analysis of common morning glory (*I. purpurea*) using the Illumina hiseq2000L sequencing yields and quality filtering.

Treatments	Sample	Reads (M)	HQ (M)	HQ (%)
Glyphosate Susceptible	068-1-1	65.8	62.9	95.6
	077-1-2	67.4	64.4	95.5
	079-1-3	41.2	39.0	94.7
Glyphosate Resistant	336-1-2	152.0	146.9	96.6
	351-1-2	57.5	54.2	94.3
	358-1-3	78.3	75.0	95.8
Total raw reads (millions)		462.2	442.4	
Average per sample		77.0	73.7	95.4
