# Supplementary material for: De Novo Assembly and Annotation of the Transcriptome of the Agricultural Weed Ipomoea purpurea Uncovers Gene Expression Changes Associated with Herbicide Resistance
Source: G3 (Bethesda). 2014 Aug 25;4(10):2035–47. doi: 10.1534/g3.114.013508 (PMC4199709; doi:10.1534/g3.114.013508)
Supplement: Supporting Information [file supp_g3.114.013508_TableS3.pdf]

Table S3 Efficiency of mapping Illumina hiseq2000 RNA-Seq reads against the *I. purpurea* reference transcriptome, a de novo cDNA assembly. Note Bowtie2 reports on paired reads, effectively halving the absolute raw and HQ read counts seen in Table S1.

Treatments	Sample	Mapped Reads (M)	Mapped Reads (%)	Unique Alignments (M)	Unique Alignments (%)
Glyphosate Susceptible	068-1-1	14.9	47.4	12.8	40.6
	077-1-2	15.1	46.8	12.8	39.6
	079-1-3	9.1	46.5	7.6	39.1
Glyphosate Resistant	336-1-2	33.5	45.6	28.7	39.1
	351-1-2	14.6	65.7	13.6	54.7
	358-1-3	22.2	59.1	19.0	50.6
Total mapped reads (millions)		109.4		94.5	
Percent of HQ reads mapped		49.4		42.8	
Percent of raw reads mapped		47.4		40.8	
