# Supplementary material for: De Novo Assembly and Annotation of the Transcriptome of the Agricultural Weed Ipomoea purpurea Uncovers Gene Expression Changes Associated with Herbicide Resistance
Source: G3 (Bethesda). 2014 Aug 25;4(10):2035–47. doi: 10.1534/g3.114.013508 (PMC4199709; doi:10.1534/g3.114.013508)
Supplement: Supporting Information [file supp_g3.114.013508_TableS4.pdf]

Table S4   Primers developed for the qPCR verification of DEGs.

Sequence Name	Sequence	Tm (50mM NaCl) C
abc-b_2016948_178F	CTT TGC TGG CTT TCT TGG AC	54.52741302
abc-b_2016948_178R	GGT GAT CGA ATG GCG TTA CT	54.99775102
atpbin-2010370_162F	CTC CGC TCT TTC TTC CAA TG	53.5183376
atpbin-2010370_162R	TGC AGT ATA TCG GTG GTG GA	55.53902069
brass-2061274_150F	CTG GTA TAA CGA GCC GGT GT	56.76005614
brass-2061274_150R	GCA TAG ATT TCG ACG GCA TT	53.33061546
ceram-2056577_177F	GAG CCA GGC TTG AGA GTG TT	57.31439485
ceram-2056577_177R	GCT GTT TGC AAT GTG AGC AT	54.56376496
cysrec-2011172_162F	GGT TCC CTA GCT CCC TCA TC	56.77992894
cysrec-2011172_162R	ACT AGG TCA CCG CCT CTT CA	57.77335135
germd-2001731_176F	TTG AGC CAA ATG GAA CAA CA	52.8778193
germd-2001731_176R	CCA AAA GTA GCC TTC CAC CA	55.03216815
glut-s_2002932_175F	TTT TGT GCA CTT GGG TTG AA	53.31376738
glut-s_2002932_175R	GCA CCA GTT TCA ATT GGC TT	54.2963209
helica-2013762_163F	TTG CAA CTG GCT TTC AAC AG	54.15438015
helica-2013762_163R	ATT TTC TGC AAA CCT GGT GG	53.78453676
P450_1-2005659_173F	TGT ATC AAC CAC GGT CTC CA	55.65938391
P450_1-2005659_173R	CGC GCT TTC CTA TCT ACC AG	55.29411425
P450_2-2003522_172F	GAG CAA AAA CCT TGC AGA CC	54.76344105
P450_2-2003522_172R	AAT TGC TGG ACA CCA ACC TC	55.56952121
P450a_2003581_168F	AGT GCT GGT GGT TAG CGA CT	58.78593189
P450a_2003581_168R	ATA AGT TTG CGA ATC CCA CG	53.24697226
pecmet_2017152_161F	TAG AAT TGC CGC TGA CTG TG	55.20539275
pecmet_2017152_161R	GAT GGG TTC ATA GCC CAA GA	54.40150801
proto_2011804_157F	CGG AGT TCT GGT ATT GGG GT	56.64581809
proto_2011804_157R	CAT AAA GGT GGC GAC GAT GG	56.10508724

serthre-2055046_161F	GGA GAA GGG AAG TCT CGA CC	56.78871641
serthre-2055046_161R	TGA TCG GAG TGT CCA ATG AG	54.20362614
vichyd_2063945_178F	TAT TTG GTG ATC GCG TGA AA	52.35251201
vichyd_2063945_178R	TGA GCA AGA AGC AAA TGG TG	53.72764614
wb-abc_2017606_160F	AAG CTT CTG TTC CTG GAC GA	56.15266566
wb-abc_2017606_160R	GGC ACA GGC TAG TGA AGA GG	57.64456754
