# Supplementary material for: De Novo Assembly and Annotation of the Transcriptome of the Agricultural Weed Ipomoea purpurea Uncovers Gene Expression Changes Associated with Herbicide Resistance
Source: G3 (Bethesda). 2014 Aug 25;4(10):2035–47. doi: 10.1534/g3.114.013508 (PMC4199709; doi:10.1534/g3.114.013508)
Supplement: Supporting Information [file supp_g3.114.013508_TableS5.pdf]

Table S5 Differentially expressed genes as identified by edgeR after binning scaffolds based on length (bins were defined as 200-1000 bp, 1001-2000 bp, 2001-3000 bp, and >3000 bp). Note resistant/susceptible log2 fold changes.

rank	bin (bp)	Scaffold ID	topblast	logFC	logCPM	PValue	padj
1	1,001-2,000	2009240	protein	-4.956514395	4.417191331	1.60E-13	1.33E-09
2	1,001-2,000	2063945	vicianin hydrolase-like	-2.951166513	5.431831016	8.98E-10	3.72E-06
3	1,001-2,000	2001731	(-)-germacrene d synthase	4.77309533	3.683966828	7.52E-09	2.07E-05
4	200-1,000	2056577	ceramidase family protein	-7.490398504	3.019096973	5.90E-09	4.79E-05
5	1,001-2,000	2009241	protein	-4.183410284	3.563076107	4.15E-08	8.60E-05
6	1,001-2,000	2005233	(-)-germacrene d synthase	5.375616041	2.268376148	9.28E-08	0.000153718
7	1,001-2,000	2010370	atp binding	-4.830721823	4.01726904	1.23E-06	0.001701055
8	1,001-2,000	2002437	protein	2.476840484	5.144280666	3.69E-06	0.004362936
9	1,001-2,000	2061274	brassinosteroid insensitive 1-associated receptor kinase 1	-4.079306873	4.148482421	4.98E-06	0.005147925
10	200-1,000	2003581	cytochrome p450 82a3-like	3.295661347	3.632279277	2.64E-06	0.010717398
11	1,001-2,000	2004377	protein	-3.861554692	3.112662587	3.24E-05	0.029797255
12	1,001-2,000	2013762	u5 small nuclear ribonucleoprotein helicase	5.089691419	1.196586827	3.61E-05	0.02985129
13	1,001-2,000	2009597	atp binding	-1.930162869	8.43404822	4.09E-05	0.030796464
14	1,001-2,000	2017152	pectin methylesterase	-3.254112192	4.114218356	4.61E-05	0.031807096
15	200-1,000	2054556	protein	3.437708896	2.968028123	1.75E-05	0.036093279
16	200-1,000	2059855	protein kinase	4.123441785	4.96632482	1.78E-05	0.036093279
17	1,001-2,000	2010065	indoleacetic acid-induced-like protein	-2.027993775	4.890619041	5.67E-05	0.036114335
